# Phyto-toponyms of *Arbutus unedo* L. and their distribution in Sardinia (Italy)

**DOI:** 10.1371/journal.pone.0181174

**Published:** 2017-07-13

**Authors:** Claudia Pinna, Luisa Carta, Vitale Deiana, Ignazio Camarda

**Affiliations:** Department of Agriculture, University of Sassari, Viale Italia, Sassari, Italy; Austrian Federal Research Centre for Forests BFW, AUSTRIA

## Abstract

The study shows the results of an inventory of place names connected to *Arbutus unedo* L., a Mediterranean species, widespread throughout Sardinia. The main aim was to compare the past distribution of place names, referring to the strawberry tree, to the current distribution of the species on the island. In addition, we investigated the meaning and the diversity of these local place names in the various communities. The result was a collection of 432 phyto-toponyms. 248 of them were used for an analysis of their distribution in the habitats, indicated on the Map of the Nature System in Sardinia, defined on the basis of the current vegetation typology. The persistence of the species in the various habitats was either confirmed or negated with in site investigations and interviews. 47.5% of municipalities have place names related to the strawberry tree. Of the 248 phyto-toponyms, 127 fall in the habitats where the species currently persists proving a correspondence between their regional distribution and the current distribution of the species. The remaining 121 phyto-toponyms fall in habitats where the strawberry tree is currently absent. Most of them are found in man-made habitats where man has transformed the forest cover which previously included the strawberry tree. This study also contributes to promoting and conserving the linguistic heritage of local communities.

## Introduction

The place names are closely related to physical [1, 2, 3, 4], biological [5, 6] and cultural features [7, 8] as evidenced by a careful reading of the geographical maps [9]. They provide a real and objective description of a landscape, and indicate precisely and unambiguously the recurrence of certain landmarks within an area, supplying precious information on its natural resources [10, 11, 12]. In fact, human communities, which over generations have settled there, have inspected it in depth, crisscrossed it and have evaluated all the numerous aspects of its geomorphology, soil, water, rivers, ponds, rocks, plants and animals. A large number of place names are also associated with human activities: work [13], agriculture [14, 15], livestock [16, 17] and beekeeping [18].

The place names are a historical testimony of a territory, which is in continuous transformation, and help to inform us about the past environmental conditions of an area [19]. They are an important starting point for history and nature studies aimed at evaluating the evolution of a landscape [20] and the past distribution of plants or animals. The place name: *Nuraghe Niu Abila* (= Nuraghe of Eagle's Nest), in the municipality of Villanova Strisaili (central Sardinia) for example, indicates a nesting site of the eagle, used in the past but non-existent today.

The names of a site are often very old and some of them have remained unchanged for thousands of years, recalling languages of the past and offering specific information on a place. The place name *Sorabile* near the village of Fonni (central Sardinia) for instance, which exists to this day, appears in a Latin inscription dating back to the first century AD as "*Nemoris Sorabensis*", which means "Forest of Sorabile" and indicates the presence of a forest in the area [21]. Interpreting place names is often difficult because they usually have a clearly defined meaning at the time of naming but may lose their transparency over time due to changes in local languages, to name distortion by oral transmission or to changes in the original feature that inspired the name. References to events that occurred in the past, but of which there is no trace today, are rather frequent in place names [22]. The place name: *Pala Brusiada* (= burned slope) near the village of Bolotana (central Sardinia) for example, covered in forest today, refers to a great fire that occurred at the beginning of the last century and is still present in the collective memory. Sometimes the meaning of place names may be completely unknown and not even the work of linguists and philologists can shed light on a plausible meaning, which, at times, still remains totally obscure. There are many place names with ambiguous meaning in Sardinia, such as *Azzani*, near the village of Talana (central Sardinia), which is considered to be of pre-Roman origin [23] and therefore indigenous to ancient Sardinian populations.

Place names associated with plants (phyto-toponyms) are among the most numerous of all. This is due to the strong link that ancient man had with the territory he inhabited. Plants were the prime and first element that he noted and on which he vitally depended. Plants, as opposed to animals, are fixed points in a landscape that can be used to identify a place immediately [24]. Therefore, plants help to define the landscape and many phyto-toponyms express the physiognomy and environmental features of an area very effectively and also testify to the present, past, even remote past, existence of these characteristics [22, 25, 26]. The place name *S’Ortu mannu de is Olias de is Pisanos* (= The Large Olive Tree Garden of the Pisans) is a place with many century-old olive trees that date back to the time of the Pisan domination of Sardinia, in the Middle Ages [27].

The identification of wild plants in one’s own territory was essential in the past for many reasons: for nourishment and fodder; recognition of poisonous crop and pasture land weeds; for medicinal, magical and artisanal purposes and most community members possessed this knowledge. In Sardinia, more so than in other regions of Italy, the relationship between man, plant and environment has always been very strong because of the island’s main economic activity of agro-silvo-pastoralism [28].

Phyto-toponyms are certainly related to the presence of plant species and to their distribution, but not necessarily to their profusion [29]. In fact, local communities, when naming a place, may have referred either to a maquis or forest and therefore to the presence of many specimens of the same species or even to a rare plant species that, through the presence even of a single specimen, could identify a place with certainty, or a single tree which, due to its size, constitutes an unequivocal landmark in the territory [30, 31].

In Sardinia out of approximately 100,000 place names listed by Paulis [23, 32], around 20,000 are attributable to, or directly related to, plants [31, 33]. Further studies carried out, at municipality level, by linguists and local experts [30, 34, 35, 36, 37, 38, 39] attest to much higher numbers—over 30,000 [40].

Place names on the island are reported in Sardinian with all the local dialectical variations of the different villages. They are often found on local signs used on tourist and nature trails.

### The Sardinian language

Knowledge of the local language is essential for a full understanding and analysis of place names, which “contain a treasure of ancient language elements which allows them to under build their theories or test their hypotheses*”* [41].

The Sardinian regional law n° 26 of the 15th October 1997 [42] confers on the Sardinian language the same *status* as Italian and recognizes four linguistic variants within the language: Campidanese, Gallurese, Logudorese, and Sassarese with their related dialects and also languages such as Catalan in the town of Alghero (western Sardinia) and Genoese on the island of San Pietro and the village of Calasetta (southwestern Sardinia). The *status* of minority language is also recognized by Italian law (n°482/1999) and by the European Union. Sardinian is a neolatin/romance language, like Italian, French, Spanish. It has been influenced by many languages deriving from the numerous dominations of the island (Phoenician-Punic, Roman, Pisan, Genoese, Catalan-Aragonese, Piedmontese) prior to the entry of Sardinia into the Kingdom of Italy. Many words can be attributed to the ancient indigenous language of the Sardinian people pre-dating the Roman and Phoenician-Punic periods [43, 44, 45, 46, 47, 48, 49, 50]. Most Sardinian words (82.7%) are considered to be of Roman origin; 12.1% are pre-Roman, 3.2% Italian, 0.8% Spanish, 0.6% Catalan, and 0.6% Byzantine [51]. In Barbagia of Ollolai (central Sardinia), close to 50% of words are pre-Roman (i.e. indigenous) [32]. Certain Sardinian words still retain the linguistic influence of ancient peoples the island’s inhabitants had dealings with; for example, *golostiu* linked to the Basque *gorosti/korosti* (= *Ilex aquifolium* L.) which attests to a very ancient contact with the Basque people [49]; the Sardinian words *zipiri* (= *Rosmarinus officinalis* L.) and *kuruma* (= *Ruta chalepensis* L.) are considered to be of Punic origin [23].

In addition to Italian, the majority of Sardinians speak the language of their own town or village and this has given rise to a large number of local variants. Such a varied language has resulted in great variations of names even in the plant kingdom [52, 53] and consequently in phyto-toponyms.

### Botanical place names and the strawberry tree

Among the trees and shrubs, the most common Sardinian phyto-toponyms refer to the evergreen oak (*Quercus ilex* L.), downy oak (*Quercus pubescens* Willd.), cork oak (*Quercus suber* L.), alder (*Alnus glutinosa* (L.) Gaertner), wild olive (*Olea europaea* s.l.), strawberry tree (*Arbutus unedo* L.), lentisk (*Pistacia lentiscus* L.). The reference may be to isolated plants (e.g. *Sa Chessa ruja* = red lentisk = *Pistacia terebinthus* L.; *Su lidone* = strawberry tree = *A*. *unedo* L.), or to forest or maquis (e.g. *Sueredu* = cork oak forest = *Q*. *suber* L.; *Chessargiu* = lentisk maquis = *P*. *lentiscus* L.; *Lidonargiu* = strawberry maquis = *A*. *unedo* L.; *S'Aliderrargiu* = phillyrea maquis = *Phillyrea latifolia* L.) [31].

*A*. *unedo* L. (*Ericaceae*) is widespread along the Mediterranean basin and also over some coastal areas with a temperate Atlantic climate in Portugal, Spain, France and Ireland [54, 55, 56]. Its most northerly limits are the cliffs from Trieux to Paimpol in northwestern France and in the state-owned land of Muckross on Briekeen Island, in Ireland, where it is the dominant vegetation [57]. Whether the strawberry tree is indigenous to Ireland has long been a subject of discussion among specialists [58], since the species has not been found in its fossilized state there; neither has its pollen been discovered in the peat-like deposits of Killarney. Of particular interest are the studies [57, 58, 59, 60, 61, 62] conducted on the phyto-toponyms of the strawberry tree recorded in the counties of Kerry, in Waterford (eastern Ireland), Clare (western Ireland) and Mayo (northwestern Ireland). The boundaries of the county of Mayo are more than 100 km north of the strawberry tree’s current limits [57], which would presume a greater distribution of this species in the past.

Place names referring to the strawberry tree are reported in other countries along the Mediterranean basin. These include, among others: Spain with the southwestern provinces of Seville and Huelva, Segovia and Ciudad Real, respectively north and south of Madrid [63], in the central province of Castilla-La Mancha [64], Aragon, in the northeast on the border with France near the Pyrenees [65]; France with Provence (southeast), Gascony (southwest, near Spain and the Pyrenees), in Saintonge (west-central); also on the Mediterranean islands of Corsica [66, 67], as well as Sicily [68] and the Balearic Islands of Mallorca [69] and Menorca [70]. More research specifically dedicated to the place names of the strawberry tree in North Africa and in the eastern Mediterranean is needed.

This study aims:

1. to count and analyze the meaning of place names that refer to the strawberry tree;
2. to investigate the distribution of these phyto-toponyms on the island;
3. to compare the relationship between the distribution of these phyto-toponyms in Sardinia and the current distribution of the strawberry tree on the island;
4. to highlight the diversity of local names relating to the strawberry tree in the various communities.

## Materials and methods

### Study area and study species

This study was undertaken in Sardinia, an island which is part of southern-central Italy, situated in the central western Mediterranean Sea (38°51'-41°15' N 8°8'-9°50' E). With a surface area of 24,100 km^2^ and a population of over 1.6 million spread out over 377 municipalities, it is the second largest Mediterranean island after Sicily.

Sardinia is approximately the centre of the area of distribution of *A*. *unedo* (*Ericaceae*), one of the most common woody mesophylous species. It is widespread throughout the island, with the exception of the top of the Gennargentu mountain range, over 1,200 m.s.l. The species, which prefers a siliceous substrata, also grows in limestone, and is an important melliferous plant of Sardinia. Bees collect the nectar of its flowers to produce the famous bitter honey known, since ancient times, for its medicinal virtues. Monumental strawberry trees are to be found scattered over the entire island [53].

### Data collection

As is the case with many Sardinian words, phyto-toponyms also differ widely from village to village (e.g. *Lidone*/*Oioi* = strawberry tree) due to the phonetic variations of the numerous dialects. For this reason, the principal Sardinian dictionaries have been consulted in order to identify the dialectical variations used in referring to the strawberry tree in Sardinia [30, 37, 71, 72, 73, 74, 75].

The following is a list of certain villages, among others, with the words most commonly used to identify the strawberry tree: *Lidone* (in Anela, Berchidda, Bitti, Bolotana, Bono, Bonorva, Ittiri, Nule, Nuoro, Orani, Ozieri, Pattada, Siniscola) and *Olidone* (in Baunei, Berchidda, Oschiri, Padria, Urzulei, Usini) are considered to be of Roman [76] or pre-Roman origin [71, 77, 78]. Alongside these, in the various dialects there are also other words: *Alboç* (in Alghero), of Catalan origin; *Armù* (in Carloforte), Genoese in origin; *Arbitru/albitru*, from the Latin *arbutus*; *Baga* (in La Maddalena), of Corsican origin; *Lioni* (in Jerzu, Perdas de Fogu, Seui, Tempio); *Oiòi* (in Mogoro); *Oiò(n)i* (in Fluminimaggiore); *Oiòni* (in Villacidro); *Oliòne* (in Laconi); *Oliòni* (in Burcei) *Orioni* (in S. Antioco).

The word *aissu*, specifically referring to the strawberry tree flower, is of uncertain etymology and may be attributable to the ancient native language in Sardinia prior to Roman domination [71]. The fruit is known as *Mela 'e lidone* or *Braghi-braghi*. These words are of unknown etymology [76].

For the inventory and identification of phyto-toponyms related to the strawberry tree, firstly the work of Paulis [23] was used, which, based on IGM (Italian Military Geografic Institute) and cadastral maps, lists the place names of the entire Region of Sardinia without giving the Italian equivalent of Sardinian words. The list was integrated with the collection of place names obtainable from the State Archives in Cagliari and also with those present in the database of Sardinian place names available on the web site of the Autonomous Region of Sardinia for which the respective coordinates have been given [79]. In all the sources consulted the place names are listed according to the municipality they belong to.

Many studies of regional territory [40] or those restricted to small areas [38, 80, 81, 82] of the island, or even to a municipality and its surrounding area [31, 34, 35, 37, 83, 84, 85, 86] have also been consulted.

For the phyto-toponyms of uncertain meaning interviews were conducted with local people who provided information about the dialectical variations, concerning the strawberry tree, present in their communities. These either confirmed or negated the exact correspondence between the place name and species under investigation.

The phyto-toponyms found were also sub-classified based on the information they provide about the landscape characteristics within the name itself, relative to the geomorphology, stream/spring of the territory, presence of man/animal shelters, roads or paths and archaeological sites. For example, the phyto-toponym *Monte su Lidone* provides two pieces of information: *Monte* meaning mountain and *Lidone* meaning strawberry tree, thus the presence of both within the same place name. A distinction was also made between those phyto-toponyms that indicate single trees and forest/maquis cover of the strawberry tree. This was possible because in Sardinian phyto-toponyms the suffixes -*ariu*, -*edu* and others, derived from Latin, with which the name of plant species can end, are used to point out that, in that particular place, the plant mentioned exists in profusion, suggesting the presence of maquis or forest related to that species [30]. The suffix -*eddu* indicates, on the other hand, the diminutive (e.g. *Lioneddu* = small strawberry tree).

Personal knowledge of our different dialects has also definitely helped us in recognizing most of the phyto-toponyms.

### Distribution of phyto-toponyms and GIS analysis

The phyto-toponyms collected were regrouped in S1–S3 Tables.

Each table presents the list of phyto-toponyms in Sardinian, their translation into English, the municipality they belong to and, when available, cartographic references, i.e. their IGM tablet number, WGS 84/UTM zone 32N coordinates for the phyto-toponyms listed in the web site of the Autonomous Region of Sardinia; or else there is a note to the effect that the phyto-toponym comes from the cadastral map. In some cases, there is no cartographic reference when the phyto-toponyms come from the State Archives list and most likely exist today only through oral tradition.

In order to indicate the distribution on the island of phyto-toponyms, even those whose coordinates were not known, a map was drawn up taking into consideration the municipality to which they belong to. Each municipality has a shade that corresponds to the number of phyto-toponyms identified. ArcGis 10.0 software was used [87].

With the phyto-toponyms for which coordinates were known, two other maps were drawn up by overlapping them with the units and the habitats indicated on the Map of the Nature System in Sardinia (scale: 1:50,000) [88, 89, 90]. The Map of the Nature System provides a key for easy and immediate recognition of the environment in relation to the units and habitats which have been defined based on the vegetation typology according to the criteria and methodology adopted by the EU and subsequently implemented by the ISPRA (The Italian National Institute for Environmental Protection and Research) for the various Italian regions. The Map of the Nature System in Sardinia enabled the identification and description of 93 habitats regrouped into 7 units (units 1, 2, 3, 4, 5, 6, 8) based on similar ecological and physiognomic-structural characteristics. Unit number 7 does not appear on this list as it includes habitats that are not present in Sardinia. Of these 93 habitats, 50 are DH (i.e. Prioritary habitats, in accordance with the EU Council Habitats Directive). The concept of habitat is the same as that adopted by the Council Directive 92/43EEC of 21 May 1992. Article 1 defines it as "terrestrial or aquatic areas distinguished by geographical, abiotic and biotic features, whether entirely natural or semi-natural" [91]. The codes of the habitats on the Map of the Nature System are the same as in Corine Biotopes [88, 89, 90].

The shape files—the punctual Map of the Distribution of those 248 phyto-toponyms of which the coordinates are known and the areal Map of the Nature System in Sardinia with the 7 units and the 93 habitats—have been overlapped, using ArcGis 10.0 software [87]. New shapefiles were thus obtained, indicating in which units and habitats the collected phyto-toponyms fall. This also enabled the assessment of the number of phyto-toponyms that fall in those units and habitats characterized by the current presence or absence of the strawberry tree as vegetation typology. In order to determine the persistence or lack of the species in the units and habitats where the phyto-toponyms fall, in site investigations were carried out as well as further interviews with local land owners, forest rangers and rural police which enabled the confirmation or exclusion with certainty of the persistence of the species.

On the maps obtained by overlapping the shapefiles, the 248 phyto-toponyms were signalled with a different symbol based on whether they fell in units or habitats where the species persists or is absent today. To render map-reading simpler, the maps show only the units and habitats where the species persists today. A GLM analysis (generalized linear model and logistic regression—family binomial) was conducted to evaluate the existence of statistically significant differences (p<0.05) in the distribution of the phyto-toponyms in the habitats. The data was analysed taking into consideration the units that show the first level of classification of the habitats. Two different models were tested: a GLM on the distribution of the 248 phyto-toponyms across the seven units and a logistic regression for the presence/absence of the phyto-toponyms in the units. R software was used [92].

## Results

### The inventory of *A*. *unedo* phyto-toponyms

The research, carried out in the entire region of Sardinia, shows the results of an inventory of phyto-toponyms related to the strawberry tree. Based on maps, database, documents and oral information, provided by local people, it was possible to ascertain that 432 phyto-toponyms, 159 more than Paulis [23], refer with certainty to the *A*. *unedo*. Of these, only 248 can be localized in the territory as their coordinates are known. Concerning the other 184 phyto-toponyms, there is information solely on the municipality they belong to (S1 Table).

The research has led to the collection of another 66 phyto-toponyms. Although it seemed that they refer to the species as they sound the same, interviews showed that:

32 of them refer to people’s names (e.g. *Cuile Lianeddu =* Julian’s sheepfold), surnames (e.g. *Casa Fortelioni* = The Forteleoni house) or to other plant genera such as *Allium*, *Alnus*, *Asphodelus* and *Olea* (S2 Table);

34 are of uncertain meaning and even unknown to persons interviewed (S3 Table).

**Fig 1:**
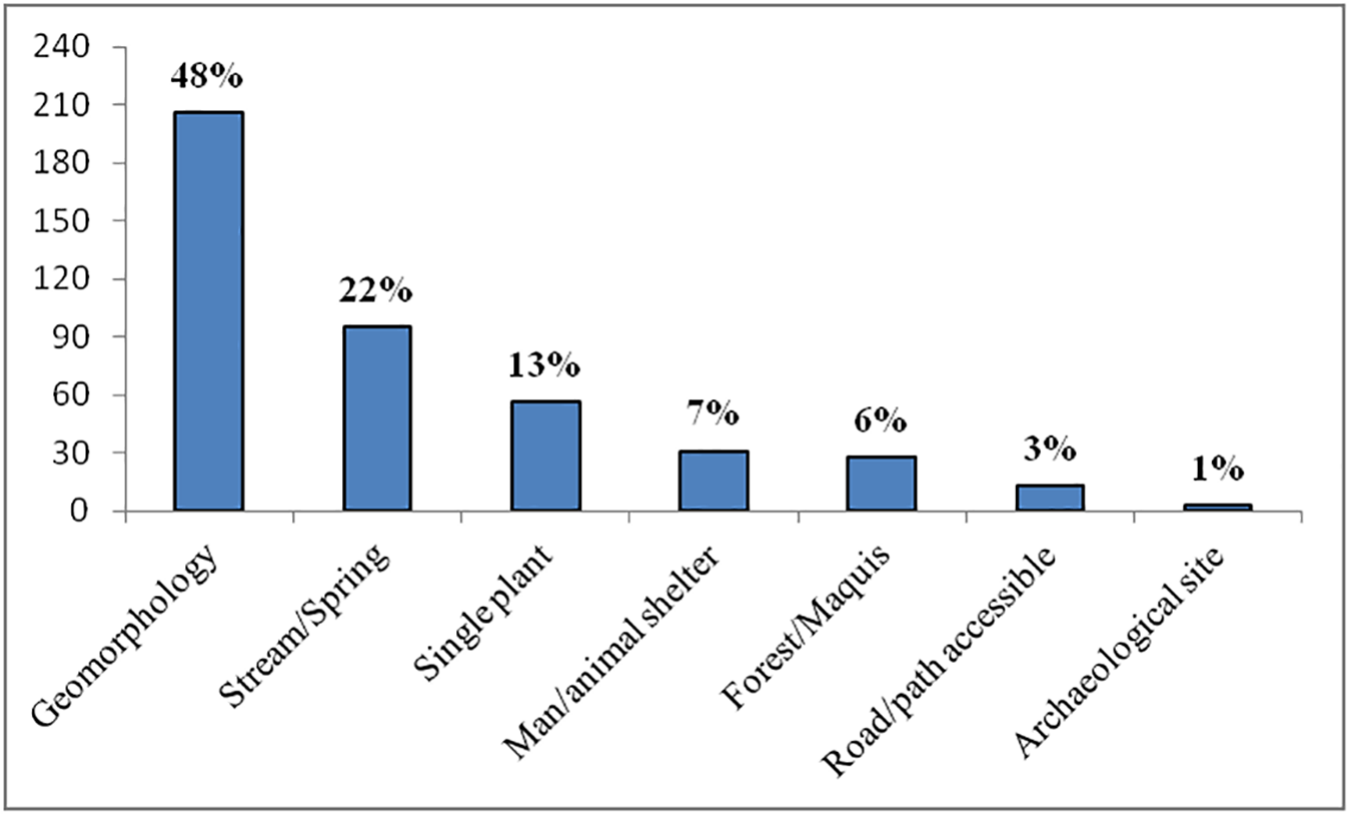
Classification of phyto-toponyms of the strawberry tree based on additional information, found within the name, concerning the landscape characteristics of the places.

In addition to the name of the plant, most of the recorded phyto-toponyms provide further information on the landscape characteristics of each place within the name itself. Fig 1 shows the classification of the phyto-toponyms based on the diversity of the information expressed.

References inspired by a place’s geomorphology and stream/spring, man/animal shelters, road or path accessible and archaeological sites occurred in more than 80% of the place names related to *A*. *unedo*. The most numerous phyto-toponyms (48%) are those that provide information on the geomorphology of a territory (e.g. *Monte S'Elidone* = Strawberry tree mountain; *Pranu de Illione =* Strawberry tree plain*; Giba S’Olioni =* Strawberry tree hill; *Pizzu Lioni* = Strawberry tree top; *Perda ‘e Lione* = Rocky place of the strawberry tree; *Sedda de Lioni* = Strawberry tree valley), followed by those (22%) that indicate the presence of water: stream/spring (e.g. *Riu su Lidone* = Strawberry tree stream; *Funtana Lidone* = Strawberry tree spring), man/animal shelters (7%) (e.g. *Casa de Lidone* = House of the strawberry tree; *CuileS’Olione =* Strawberry tree sheepfold; *Pinnetta Elidone =* Strawberry tree hut), road or path accessible (3%) (e.g. *Strada Sa Serra ‘e Lione* = Road of the strawberry tree ridge) and archaeological sites (1%) (*Nuraghe de S’Olioni =* Strawberry tree nuraghe). As for the remaining 19% of phyto-toponyms, 13% point to the presence of single strawberry trees (e.g. *Sa Matta S’Ollioni* = The strawberry tree plant) and 6% to strawberry tree maquis or forest (e.g. *Sa Lionera* = The strawberry tree forest/maquis).

### Distribution of *A*. *unedo* phyto-toponyms and persistence of the species

**Fig 2:**
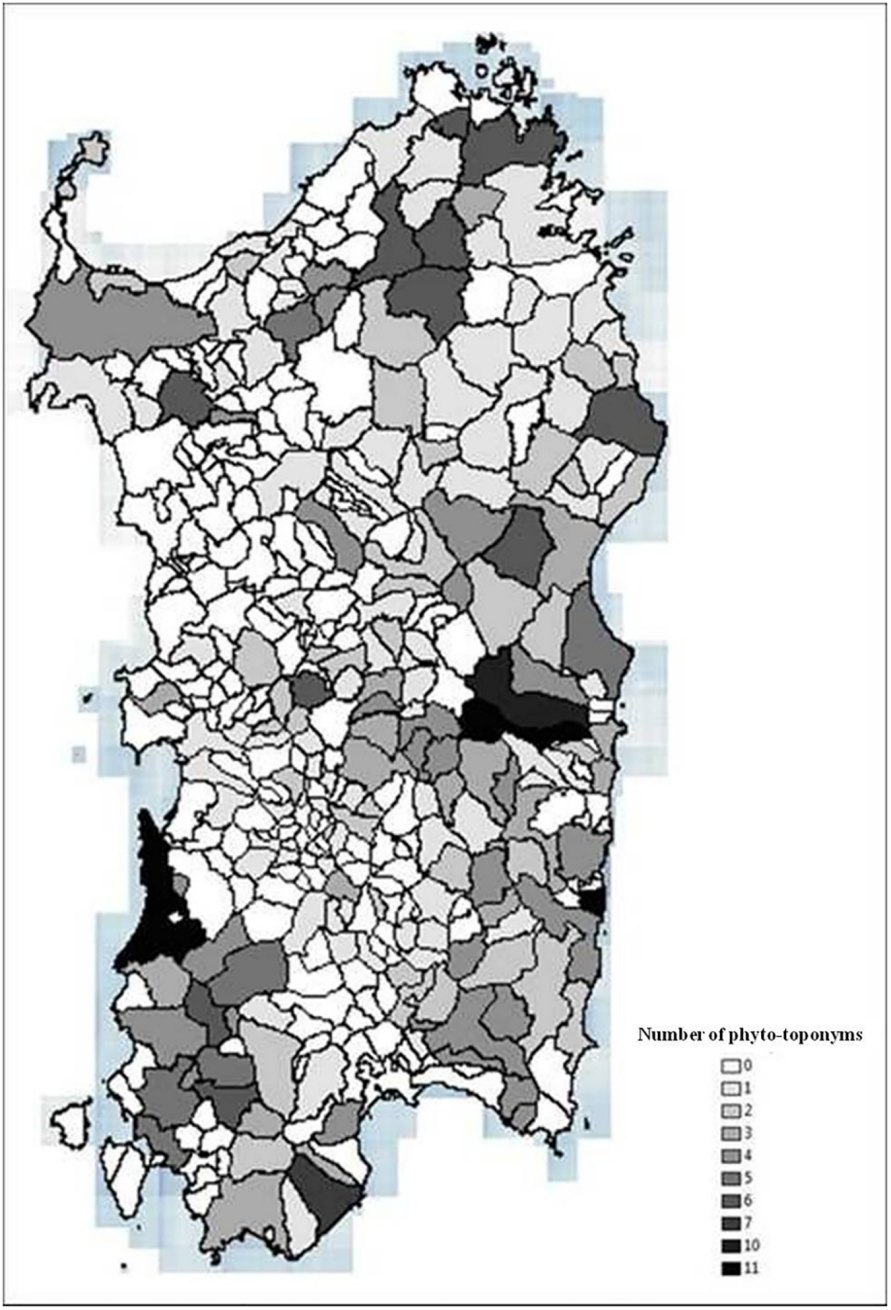
Distribution of 432 phyto-toponyms related to the strawberry tree in Sardinian municipalities.

**Fig 3:**
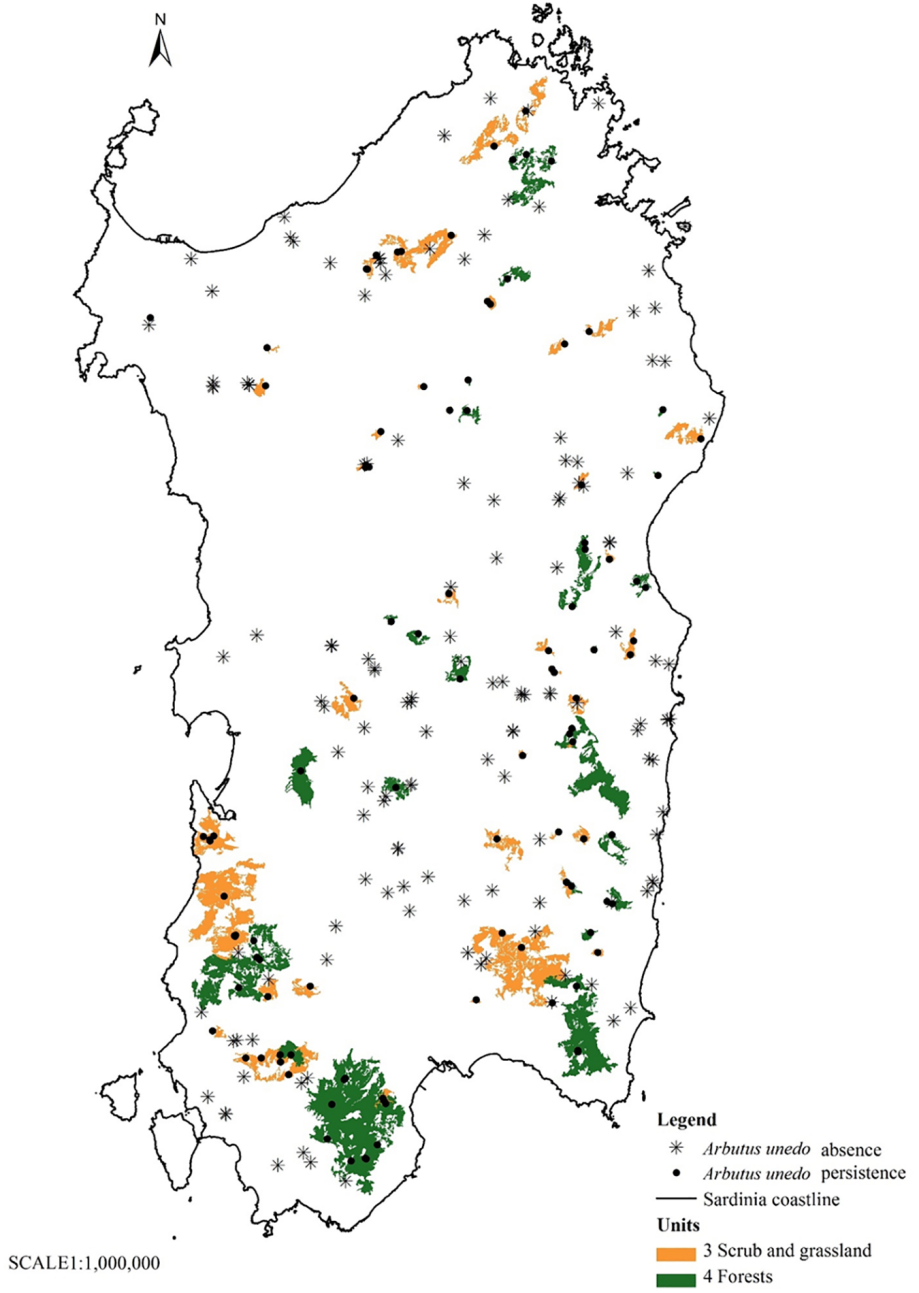
Distribution of 248 phyto-toponyms and units where the strawberry tree currently persists.

**Fig 4:**
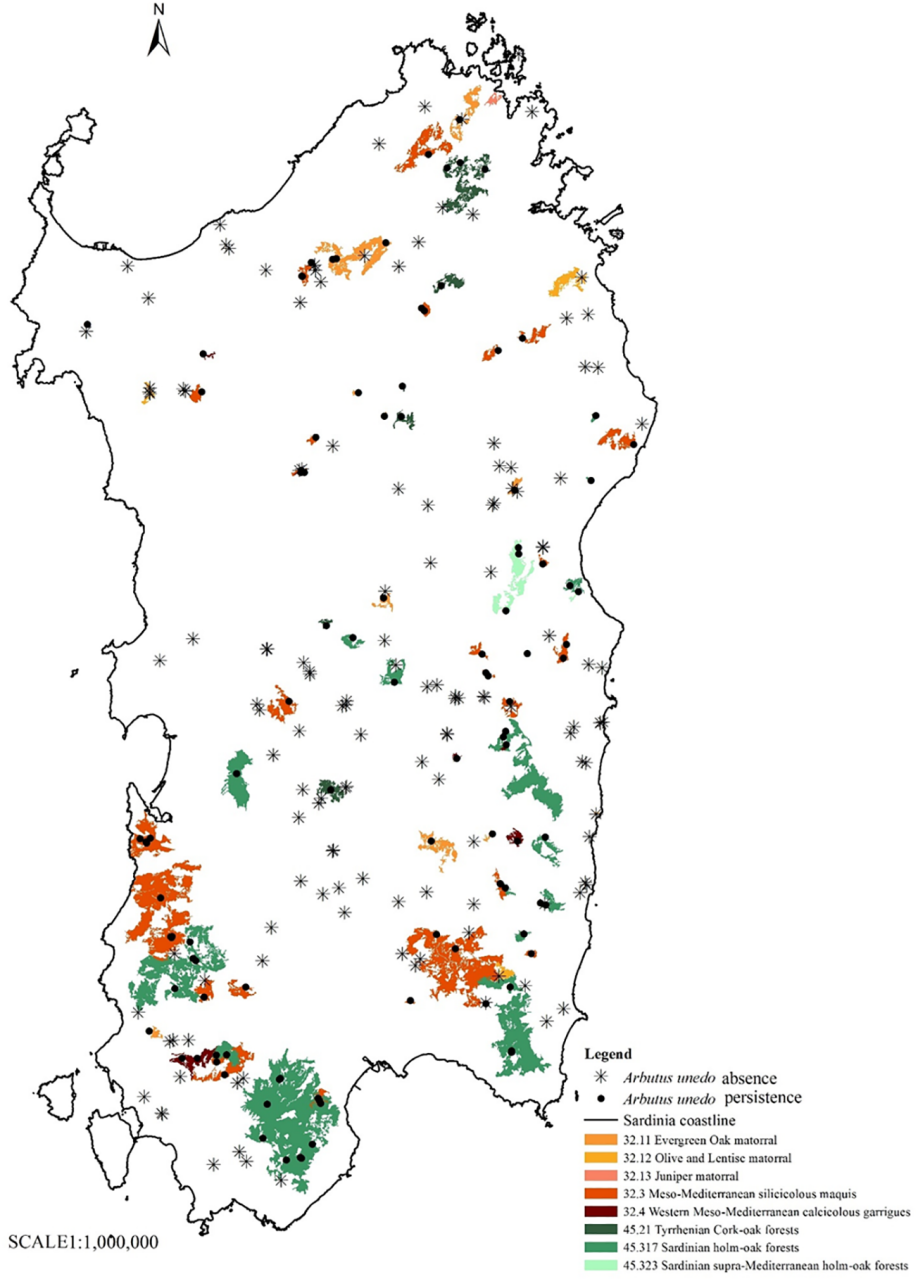
Distribution of 248 phyto-toponyms and habitats where the strawberry tree currently persists.

The map (Fig 2) shows the number of phyto-toponyms reported at municipality level. 47.5% of Sardinian municipalities have place names connected to the strawberry tree. The maps (Figs 3 and 4) indicate the distribution of the phyto-toponyms on the island and, in particular, the two units and eight habitats in which the phyto-toponyms fall and the strawberry tree currently persists as vegetation typology.

Table 1 shows that 127 of the 248 phyto-toponyms, connected to the strawberry tree, fall in those habitats, as reported on the Map of the Nature System in Sardinia, where the species currently persists. In particular, more than 65% of these fall into two habitats that are specific to the *A*. *unedo*:

51 phyto-toponyms in the habitat 32.3-Meso-Mediterranean silicicolous maquis (unit 3: Scrub and grassland) which includes maquis or forest of *A*. *unedo* and *Erica arborea* L;

32 phyto-toponyms in the habitat 45.317-Sardinian holm-oak forests (unit 4: Forests), a final stage of the succession series of the silicicolous maquis of the *Ericaceae* in the areas of siliceous substratum, from sea level up to 1000–1200 metres in altitude.

**Table 1:** List of the 7 units and 93 habitats reported on the map of the Nature system in Sardinia [88, 89, 90] with their respective codes, surface area (km^2^) of each habitat, number of phyto-toponyms and current persistence of the strawberry tree in each habitat.

Units	Code	Habitat	Area (km^2^)	Number of phyto-toponyms	Persistence of *A*. *unedo*
1. Costal and halophytic communities	15.1	Salt pioneer swards. Salicornia and other annuals DH	28.45	0	no
	15.5	Mediterranean salt meadows DH	25.71	0	no
	15.6	Saltmarsh scrubs DH	0.65	0	no
	16.1	Sand Beaches DH	16.21	1	no
	16.21	Shifting Dunes DH	2.05	0	no
	16.22	Grey Dunes DH	4.87	0	no
	16.27	Dune Juniper thickets and woods DH	19.32	0	no
	16.28	Dune sclerophyllous scrubs DH	29.38	0	no
	16.29	Woodes dunes DH	34.89	0	no
	16.3	Humid dune-slacks DH	1.50	0	no
	17.1	Unvegetated shingle beaches	0.37	0	no
	18.22	Mediterranean cliff communities DH	39.73	0	no
	19	Islets and rock stacks DH	1.66	0	no
2. Non-marine waters	21	Lagoons DH	119.13	0	no
	22.1	Fresh waters DH	109.38	0	no
	22.4	Aquatic Vegetation DH	2.10	0	no
	23	Standing brackish and salt water	1.20	0	no
	24.1	River course DH	5.71	0	no
	24.225	Mediterranean gravel beds DH	18.80	0	no
3. Scrub and grassland	31.43	*Juniperus oxycedrus* scrub DH	0.59	0	no
	31.75	Cyrno-Sardian hedgehog-heaths DH	136.82	7	no
	31.81	Medio-European rich-soil thickets	2.95	0	no
	31.844	Tyrrhenian broom fields	0.46	0	no
	31.845	*Genista aetnensis* stands	0.10	0	no
	31.863	Supra-Mediterranean bracken fields	1.84	0	no
	31.8A	Tyrrhenian sub-mediterranean deciduous thickets	0.16	0	no
	32.11	Evergreen Oak matorral DH	1179.58	11	yes
	32.12	Olive and Lentisc matorral	684.45	8	yes
	32.13	Juniper matorral DH	337.74	1	yes
	32.14	Pine matorral	15.75	0	no
	32.18	Laurel matorral DH	0.54	0	no
	32.211	Oleo-lentisc brush	1268.33	18	no
	32.212	Thermo-Mediterranean heath-garrigues	0.54	0	no
	32.215	Calicotome brush	34.97	0	no
	32.217	Coastal *Helichrysum* garrigues DH	17.65	0	no
	32.218	Myrtle thickets	12.88	0	no
	32.219	Thermo-Mediterranean kermes oak brushes	0.12	0	no
	32.22	Tree Spurge formations DH	36.27	0	no
	32.23	Diss-dominates garrigues DH	15.44	0	no
	32.24	Palmetto brush DH	0.59	0	no
	32.26	Thermo-Mediterranean broom fields (retamares) DH	5.97	0	no
	32.3	Meso-Mediterranean silicicolous maquis	2440.95	51	yes
	32.4	Western Meso-Mediterranean calcicolous garrigues	298.90	7	yes
	33.2	Sardinian *Centaurea horrida* phryganas DH	1.63	0	no
	33.9	Cyrno-Sardinian *Genista phrygana* DH	2.87	0	no
	34.326	Sub-Mediterranean mesobromion DH	24.42	0	no
	34.5	Mediterranean xeric grasslands DH	132.46	0	no
	34.81	Mediterranean subnitrophilous grass communities	3026.53	11	no
	35.3	Mediterranean siliceous grasslands DH	291.87	2	no
	38.1	Mesophile pastures	33.36	1	no
4. Forests	41.72	Cyrno-Sardinian white oak woods	312.05	7	no
	41.732	Southern Italian and Sicilian *Quercus pubescens* woods	329.86	0	no
	41.81	Hop-Hornbeam woods	2.08	0	no
	41.9	Chestnut woods DH	9.71	0	no
	41.D1	Inner Alpine Aspen woods	0.04	0	no
	42.82	Mesogean Pine forests (*Pinus pinaster*) DH	1.03	0	no
	42.83	Stone Pine forests (*Pinus pinea*) DH	8.83	0	no
	42.84	Aleppo Pine forests DH	11.83	0	no
	42.A7	Yew woods DH	1.00	0	no
	44.12	Lowland. collinar and mediterranean-montane willow brush DH	27.40	0	no
	44.13	White willow gallery forests DH	2.34	0	no
	44.61	Mediterranean riparian poplar forests DH	8.30	0	no
	44.63	Mediterranean riparian ash woods DH	56.71	1	no
	44.81	Oleander, chaste tree and tamaris galleries DH	76.32	0	no
	44.91	Alder swamp woods	0.24	0	no
	45.1	Olive-Carob forests DH	570.38	5	no
	45.21	Tyrrhenian Cork-oak forests DH	1035.97	12	yes
	45.317	Sardinian holm-oak forests DH	1950.34	32	yes
	45.323	Sardinian supra-Mediterranean holm-oak forests	225.74	5	yes
	45.8	Holly-wood DH	0.53	0	no
5. Bogs and marshes	53.1	Reed beds	58.88	0	no
	53.6	Riparian cane formations	10.81	0	no
6. Inlands rocks, screes and sands	61.3B	Central Mediterranean screes DH	0.54	0	no
	61.3C	Central Mediterranean acidophilous screes DH	1.37	0	no
	62.11	Western Eu_Mediterranean and Oro-Iberian calcareous cliffs DH	175.17	1	no
	62.24	Cyrno_Sardian montane cliffs DH	44.13	2	no
8. Agricultural land and artificial landscapes	82.1	Unbroken intensive cropland	664.59	4	no
	82.3	Extensive cultivation	3951.82	25	no
	82.4	Flooded crops	24.91	0	no
	83.11	Olive groves	595.74	8	no
	83.15	Fruit Orchards	77.20	0	no
	83.16	Citrus Orchards	53.93	0	no
	83.21	Vineyards	261.56	2	no
	83.31	Conifer plantations	945.22	16	no
	83.322	Eucalyptus plantations	219.91	1	no
	83.325	Other broad-leaved tree plantations	8.57	0	no
	84.6	Dehesa	1126.68	9	no
	85.1	Large Parks	7.57	0	no
	86.1	Towns	557.20	0	no
	86.3	Active industrial sites	116.46	0	no
	86.41	Quarries	73.13	0	no
	86.6	Archeological sites	2.09	0	no
	89	Industrial lagoons and reservoires. canals	26.54	0	no

DH = priority habitats

The remaining 121 phyto-toponyms fall in habitats where the strawberry tree is currently absent and, more specifically, 54% of these fall in unit 8 (Agricultural land and artificial landscapes) which includes man-made habitats. In particular, the phyto-toponyms fall in the following man-made habitats: 82.3 extensive cultivation (n° 25), 83.31 conifer plantations (n° 16), 84.6 dehesa (n° 9), 83.11 olive groves (n° 8), 82.1 unbroken intensive cropland (n° 4), 83.21 vineyards (n° 2) and 83.322 eucalyptus plantations (n° 1) (Table 1).

**Fig 5:**
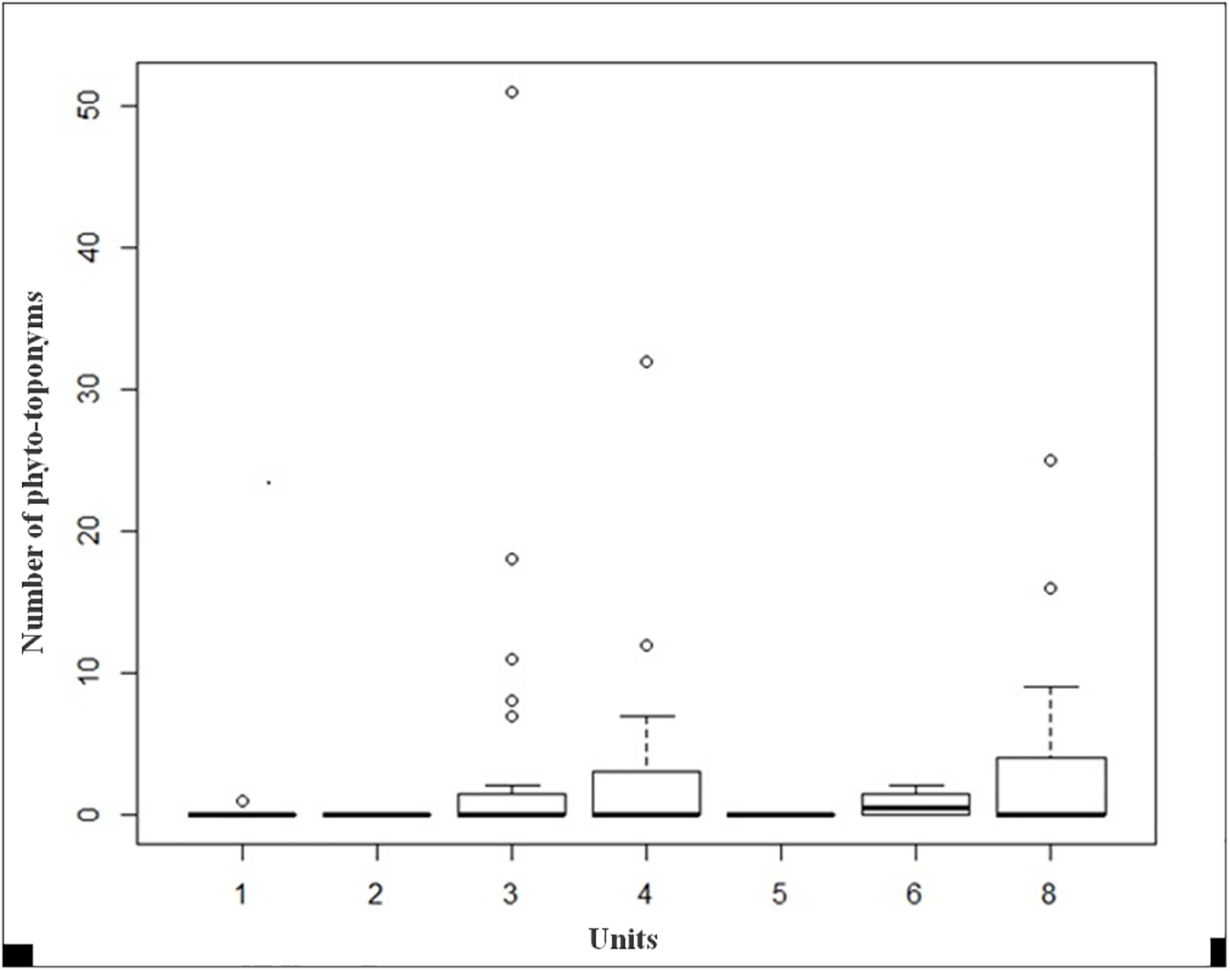
Box plot of the distribution of the 248 phyto-toponyms in the 7 units.

The first model on the distribution of the 248 phyto-toponyms across the seven units (Fig 5), tested with a generalized linear model, resulted as not significant. On the contrary, the second model on presence/absence of phyto-toponyms in the seven units, tested with logistic regression, resulted to be significant (p = 0.017; Table 2); however, only for units 6 and 8 (respectively, Inlands rocks screes and sands p = 0.085 and Agricultural land and artificial landscapes p = 0.065; Table 2) we were able to reject the null hypothesis of random distribution across the units.

**Table 2:** Results of GLM (logistic regression—family binomial) on the presence/absence of the 248 phyto-toponyms across the 7 units.

Coefficients:	Estimate	Std. Error	z value	Pr(>|z|)	Signif. codes
Intercept	-2.485	1.041	-2.387	**0.017**	*
unit 2	-15.081	1615.104	-0.009	0.992	n.s.
unit 3	1.743	1.109	1.571	0.116	n.s.
unit 4	1.638	1.15	1.425	0.154	n.s.
unit 5	-15.081	2797.442	-0.005	0.996	n.s.
unit 6	2.485	1.443	1.722	**0.085**	**.**
unit 8	2.128	1.152	1.848	**0.065**	**.**

Signif. codes: n.s. = not significant

***’ 0.001

‘**’ 0.01

‘*’ 0.05, ‘.’ 0.1

Deviance Residuals: Min = -1.177, 1Q = -0.883, Median = -0.845, 3Q = 1.332, Max = 2.265

Unit 6 includes habitats on particularly arid limestone substrata and on high mountain areas at the limit of strawberry tree distribution; unit 8 regroups highly man-made habitats. The strawberry tree does not currently persist in either of these two units (Table 1).

### Diversity of local names relating to the strawberry tree

This study has brought to light the existence of many variations in dialect that refer to the strawberry tree in phyto-toponyms, such as: *Ali(d)doni*, *Elidone*, *Elione*, *Elioni*, *Illione*, *Leone*, *Lidone*, *Lidoni*, *Lione*, *Lioni*, *Obioi*, *Oglioni*, *Oioni*, *Ol(l)ione*, *Ol(l)ioni*, *Olidone*, *Orioni*, *Ulidone*, *Ulione* and *Ulioni*. *Arbuticci*, in the municipality of La Maddalena, is clearly of allochthonous origin, as this community was on the whole strongly influenced by non-Sardinian peoples. Not all words referring to the strawberry tree that are in dictionaries or are used in the spoken language were found in the phyto-toponyms collected. Some examples of this are: *Alboç Armù*, *Arbitru/albitru*, *Baga*, *Braghi-braghi* and *Mela 'e lidone*.

The diminutives of terms that refer to the strawberry tree also frequently appear in the phyto-toponyms: *Bilidoneddu*, *Leoneddu*, *Lioneddu* and *Olioneddu*, which mean “small strawberry tree or tiny strawberry tree” and *Lidonargeddu*, *Lionedu* and *Lionitzos*, which mean “small strawberry tree forest/maquis”.

Depending on the local dialect of a particular area, a maquis or forest of strawberry trees is indicated with different endings–*argia*,*–agliu*,*–alzu*,*–argiu*,*–ariu*,*–arzu*,*–axiu*,*–azu*,*–aglia*,*–edu*,*–era* in phyto-toponyms such as *Alionargia*, *Lidonagliu*, *Lidonalzu*, *Olidonalzu*, *Lidonargiu*, *Olionargiu*, *Lionargiu*, *Lidonariu*, *Lidonarzu*, *Oglionaxiu*, *Lidonazu*, *Lionaglia*, *Liunaglia*, *Lionedu*, *Lionera*.

Even within the same community there are phyto-toponyms that point to strawberry trees in different ways (e.g. *Olioni/Oioni* in the municipality of Arbus, *Lidone/Olidone* in the municipality of Oliena, as well as to the maquis or forest of strawberry trees (e.g. *Lionaglia/Lidonalzu* in the municipality of Erula, e.g. *Lionitzos/Lionera* in the municipality of Aritzo. These may be the result of erroneous transcription or of existing variations within the community itself.

The name of the plant is often also accompanied by a descriptive word: *Casa S’Olioni Mannu* (House of the big strawberry tree), *Riu Gutturu di Mannolioni* (Stream of the lane of the big strawberry tree), *Domos lu Lione Toltu* (Houses of the crooked strawberry tree) and *S’Olidone Longu* (The tall strawberry tree).

The research also showed that a phyto-toponym may undergo change over time. This is the case of *Erillione*, indicated for the municipality of Tonara (central Sardinia) which, according to the local experts, was once known as *I riu Illione*, and refers to the presence of the strawberry tree in the vicinity of a stream; and similarly, the case of *Irove Olidone*, in the municipality of Baunei, whose correct form is *Giroe Olidone* and means a place surrounded by strawberry trees. This may be the result of an erroneous interpretation or transcription on the part of the map editors or to name distortion during oral transmission. It was also discovered that certain phyto-toponyms indicated on the IGM 1:25,000 map are unfamiliar to the persons interviewed and that the same area is today called something else but which still refers to the strawberry tree; for example, *Cuccuru Lioni Fois* = “crown of the strawberry tree that belongs to Mr Fois”, inhabitant of San Nicolò Gerrei, is today known as *S’Olionaxeddu* (= Small forest/maquis of strawberry tree) owing to the large number of *A*. *unedo* found there. The change in name is presumably attributable to change of propery owner. The land probably no longer belongs to Mr. Fois but the indication of the presence of the strawberry tree has been preserved in the phyto-toponym.

Other names such as *Lidene*, *Lieneddu*, *Foxiglioni*, *Oglionis*, *Tuvuglione*, *Scala di Lilioni*, *Puddelidone*, *Costa Ladolionis*, *Bau Gena Liones*, *Gurdulionis*, *Mannolioni*, *Tupulidone* among others, are most likely the result of a mistake in the transcription of the name. Consequently, the correct forms, written in italics, are indicated in the tables after a dash (e.g. Sulidone—*Su Lidone*, Erillione—*I Riu Illione*, Foxiglioni—*Foxi Lioni*); still others probably refer to the same place but were misspelled during their transcription from cadastral to IGM maps so they are written in the tables after a slash (e.g. Bruncu Elione/B.‘e Lione, Monte Selidone/M. S’Elidone, Tuvuglione/Tuvu Lione) (S1–S3 Tables).

According to Miglior [93], it seems that in Sardinia there were villages, today inexistent, that took their name from the widespread *A*. *unedo* species. These are:

*Gelidoni*, (from the Greek *ghe* = earth, and from the Sardinian *lidoni* = strawberry tree), a village near Sorso;

*Geralioni* (= strawberry tree wax?), a village in the diocese of Galtellì;

*Leonissa* or *Lionissa* in Mandrolisai near the village of Atzara that disappeared around the 15th century. Although there are no remains of the settlement, it can easily be located by its place name, which still reports this name, or by the still existing church of Santa Maria (*Santa Maria ‘e susu*) [94];

*Onevola* near Anela (from *unedula* = *unum edo*?), a specific epithet of the species *A*. *unedo*.

References to the strawberry tree are found today in the names of some villages such as *Is Lionis* (= The strawberry tree forest/maquis), near San Giovanni Suergiu and *Lu Lioni* (= The strawberry tree) in Padru and even in certain Sardinian surnames such as *Lioni* and the less common *Orioni* in Narcao and Nuxis, villages of southwestern Sardinia.

## Discussion

Toponomy is a rich source of information and a fundamental element for the understanding of a natural environment and of the landscape of a geographically isolated region like Sardinia, with its many and varied habitats and dialects. However, any analysis of place names necessitates a solid linguistic basis especially in the case of a region like Sardinia with its various dialects that change from one village to another. Consequently, research carried out, in the entire region of Sardinia, on place names connected to the strawberry tree, first required consulting the principal dictionaries of Sardinian. Familiarity with the language and the various dialects as well as interviews with local experts were fundamental in conducting a reliable inventory of phyto-toponyms in order to identify those which were erroneously transcribed on consulted maps and documents but which nevertheless referred to the strawberry tree, to point out those of uncertain meaning and to eliminate others which, from a phonetic point of view, may have been attributed to the species.

Research, conducted using the various sources, led to the identification of 432 phyto-toponyms. The numerous dialect variations, not all of which are found in the place names that refer to the strawberry tree, and, in particular, those that end with the suffix from the Latin–*arium* to indicate a place where the plant grows in profusion (maquis or forest), further enhance the study of recorded phyto-toponyms.

The research pointed out that 127 of the 248 phyto-toponyms fall in habitats of current persistence of the species. In particular, more than 65% of the phyto-toponyms related to *A*. *unedo* actually overlap with its specific habitats: silicicolous forest/maquis and Sardinian holm-oak forests. The latter habitat is the final stage of the succession series of the silicicolous maquis of the *Ericaceae* consisting in Sardinia of *A*. *unedo* and *E*. *arborea*. In fact, the natural evolution of the *E*. *arborea* and *A*. *unedo* maquis is toward the holm-oak forest. As the maquis of the *Ericaceae* gradually evolves to the holm-oak forest, the *E*. *arborea*, a species more heliophilous than the strawberry tree, progressively tends to disappear. The strawberry tree, thanks to its greater resistance to shade, on the other hand, is able to persist as a large tree within or along the edges of the holm-oak forest, testifying to this natural succession. As to the 121 phyto-toponyms that actually overlap with the habitats where *A*. *unedo* is currently absent, 54% of them fall in habitats characterized by the current presence of farm land, olive groves, vineyards, pasture land, or reforestation with non-native species. This points to the fact that the current landscape has undergone considerable change through human activity that, over the years, has affected the pre-existing vegetation and led to the disappearance of the strawberry tree while the corresponding phyto-toponyms have remained. At the same time, rural abandonment has determined the natural evolution of vegetation which has in turn influenced the current distribution of the strawberry tree on the island.Therefore, the current distribution of the species on the island may be the result of natural evolution and/or anthropic transformation of the forest cover.

Statistical analysis showed no significant differences in the abundance of the 248 phyto-toponyms across the seven units of habitats. However, the presence/absence pattern of phyto-toponyms in the seven units resulted to be significant, importantly for units 6 (Inlands rocks screes and sands) and 8 (Agricultural land and artificial landscapes) that include habitats, respectively at the limit of strawberry tree Sardinian range and highly man-made habitats, where the strawberry tree does not currently persist any longer.

This type of analysis may prove to be particularly useful for the purposes of territorial management especially when dealing with priority habitats (EU Habitats Directive) [91] where the original vegetation is to be examined and restored.

A phyto-toponym, although of ancient origin, as in the case of "*Nemoris Sorabensis*" (*=* Forest of Sorabile), does not necessarily prove that, in that place, the forest has existed for 2,000 years but nevertheless it does indicate its potentiality.

At the same time the phyto-toponym certainly indicates the presence of the plant species and, even if the plant is absent today, testifies to the ecological compatibility of the place for the plant cited and for its past presence.

Moreover, phyto-toponyms, which are the most frequent category among Sardinian place names, have proven to be a precious tool for inter-disciplinary studies. They constitute an indispensable data bank, not only for environmental studies, since they persist long after the landscape changes or the plant species disappears, but also for linguists and other specialists [31, 95, 96]. Despite the fact that phyto-toponyms which refer to the same plant species have different phonetics because of the existence of many different dialects, it is possible to find their origin and meaning thanks to omeomastic sequence. This in turn allows the identification of the local variations which are due to the numerous dominations and other historic events of the island, from as far back as the nuragic civilization right up to the present time and which have influenced the evolution of the Sardinian language.

This research confirms the significance of phyto-toponymy for previous and present knowledge of the landscape, which in many cases is changing today in an irreversible way. In fact, the disappearance of a single plant or a forest/maquis does not always lead to the disappearance of the phyto-toponym [97]. At the same time, this study also renders a contribution to the preservation of the identity of places as well as to the promotion of the linguistic and cultural heritage of communities at a time of risk of disappearance of minority languages.

## Supporting information

S1 Table Phyto-toponyms related to the strawberry tree in Sardinia. (DOC)

S2 Table Place names not related to the strawberry tree in Sardinia but which, due to similar sound, seem to refer to the species. (DOC)

S3 Table Place names of uncertain meaning but which, due to similar sound, seem to refer to the strawberry tree in Sardinia. (DOC)
