# Supplementary material for: Phyto-toponyms of Arbutus unedo L. and their distribution in Sardinia (Italy)
Source: PLoS One. 2017 Jul 13;12(7):e0181174. doi: 10.1371/journal.pone.0181174 (PMC5509287; doi:10.1371/journal.pone.0181174)
Supplement: S1 Table — (DOC) [file pone.0181174.s001.doc]

**S1 Table. Phyto-toponyms related to the strawberry tree in Sardinia.**

| **Phyto-toponyms in Sardinian language** | **Meaning** | **Sources consulted** | | | | **Municipality** |
| --- | --- | --- | --- | --- | --- | --- |
|  |  | **IGM^a^** | **WS^b^** | **CM^c^** | **SA^d^** |  |
| Stazzo lu Lioni | Strawberry tree enclosure |  | E=508393  N=4544456 |  |  | Aglientu |
| S’Olidone | The strawberry tree |  |  |  | X | Aidomaggiore |
| S’ Elidone | The strawberry tree | 194 I NE | E=534880  N=4498543 |  |  | Alà dei sardi |
| Lione | Strawberry tree |  |  | X |  | Alghero |
| Bruncu Lioneddu / Leoneddu | Rocky outcrop of the small strawberry tree |  |  |  | X | Allai |
| Leonedu | Small strawberry tree forest/maquis | 217 I NE | E=488332  N=4420478 |  |  |  |
| Casa S’Olioni Mannu | House of the big strawberry tree | 225 IV NO | E=456590  N=4389250 |  |  | Arbus |
| Canale S’Olioni | Hollow of the strawberry tree | 225 IV NO | E=455215  N=4389937 |  |  |  |
| Cuccuru de Genna Olioni | Crown of the strawberry tree pass |  |  |  | X |  |
| Cuccuru de S’Ena S’Olioni | Crown of the strawberry tree spring |  |  |  | X |  |
| Cuccuru S’Olioni Mannu | Crown of the big strawberry tree |  |  |  | X |  |
| Ena S’Olioni | Strawberry tree spring |  |  |  | X |  |
| Genna S’Oioni | Strawberry tree pass | 225 IV SO | E=459805  N=4376784 |  |  |  |
| Is Olionis | The strawberry trees (forest/maquis) |  |  | X |  |  |
| Meddu de S’Ena de S’Olioni Mannu | ? of the big strawberry tree spring |  |  |  | X |  |
| Monte S' Olioni Mannu | Mountain of the big strawberry tree | 225 IV NO | E=456704  N=4389007 |  |  |  |
| Riu is Olioneddus | Stream of the small strawberry tree forest/maquis |  | E=457500  N=4390080 |  |  |  |
| Bilidoneddu | Small strawberry tree |  |  | X |  | Ardauli |
| Funtana Lidone | Strawberry tree spring | 207 III NO | E=496664  N=4437341 |  |  |  |
| Funtana S’ Illione | Strawberry tree spring |  |  | X |  | Aritzo |
| Punta Lionitzos | Peak of the small strawberry tree forest/maquis | 218 I NO | E=519093  N=4423736 |  |  |  |
| Rio Funtana Olioni | Stream of the strawberry tree spring |  |  | X |  |  |
| Sa Lionera | The strawberry tree forest/maquis | 218 I NO | E=521255  N=4424107 |  |  |  |
| S’accu de Soioni *- S’accu de S’Oioni* | The gorge of the strawberry tree |  | E=536443  N=4379013 |  |  | Armungia |
| Baddu Lione | Strawberry tree ford |  |  |  | X | Arzachena |
| Lione | Strawberry tree | 168 II NO | E=526814  N=4549933 |  |  |  |
| Sarra di lu Lioni - *Serra di lu Lioni* | Strawberry tree ridge | 168 II NE | E=532992  N=4553871 |  |  |  |
| Scala di lu Lioni | Steep climb of the strawberry tree |  |  |  | X |  |
| Sorgente lu Lioni | Strawberry tree spring |  | E=542403  N=4551544 |  |  |  |
| Stazzo lu Lione | Strawberry tree enclosure | 168 II NO | E=526293  N=4549886 |  |  |  |
| Alionargia | Strawberry tree forest/maquis |  | E=554539  N=4379672 |  |  | Arzana |
| Baccu Lione | Strawberry tree gorge |  |  | X |  |  |
| Bruncu Lione | Rocky outcrop of the strawberry tree | 218 I NE | E=537408  N=4420432 |  |  |  |
| Bruncu (S’) Olioni | Rocky outcrop of the strawberry tree |  |  | X |  |  |
| Cuccuru Illione | Strawberry tree crown |  | E=525364  N=4421619 |  |  |  |
| Pranu de Illione | Strawberry tree plain | 218 I NO | E=526076  N=4421221 |  |  |  |
| Riu Illione | Strawberry tree stream |  | E=525338  N=4421088 | X |  |  |
| Riu s’Alionargia | Stream of the strawberry tree forest/maquis |  | E=554237  N=4380189 |  |  |  |
| Serra 'e Lione | Strawberry tree ridge | 218 I NE | E=531612  N=4421606 |  |  |  |
| Strada Comunale Bruncu Lione | Local road of the rocky outcrop of the strawberry tree |  | E=537717  N=4419364 |  |  |  |
| Strada Sa Serra ‘e Lione | Road of the strawberry tree ridge |  | E=531851  N=4421359 |  |  |  |
| Funtana Lioni | Strawberry tree spring |  |  | X |  | Atzara |
| Lioneddu | Small strawberry tree |  |  | X |  |  |
| Riu ‘e Lione | Strawberry tree stream | 218 IV NO |  | X |  |  |
| Roja Lieneddu | Stream of the small strawberry tree |  |  | X |  |  |
| Funtana Lidone | Strawberry tree spring | 207 III SE | E=509647  N=4434044 | X |  | Austis |
| Acqua S’ Olioni | Strawberry tree water |  | E=535350  N=4379869 | X |  | Ballao |
| Rio Acqua Olioni | Stream from the strawberry tree water |  |  | X |  |  |
| Sa Mitza ‘e S’Ollioni | The strawberry tree spring | 226 I SE | E=535292  N=4379928 |  |  |  |
| Casa Genna Lione | House of the strawberry tree pass |  | E=554343  N=4406798 |  |  | Barisardo |
| Genna Lione | Strawberry tree pass |  |  | X |  |  |
| Riu Gennalioni - *R.* *Genna Lioni* | Stream of the strawberry tree pass |  | E=553593  N=4407094 | X |  |  |
| Irove Olidone - *Giroe Olidone* | Place surrounded by strawberry trees |  | E=552722  N=4444866 |  |  | Baunei |
| Genna Olidone | Strawberry tree pass | 208 III SE | E=550047  N=4433126 |  |  |  |
| Nuraghe Genna Olidone | Nuraghe of the strawberry tree pass | 208 III SE |  |  |  |  |
| Olidone | The strawberry tree |  |  | X |  |  |
| Serra S’Olidone | Strawberry tree ridge | 208 III NE | E=550781  N=4446228 |  |  |  |
| Solidone Longu - *S’Olidone longu* | The tall strawberry tree | 219 IV NE | E=557984  N=4427930 |  |  |  |
| Perda Lione | Rocky place of the strawberry tree | 218 IV NE | E=511822  N=4424733 |  |  | Belvì |
| Su Lidone | The strawberry tree | 194 IV SE | E=513329  N=4483873 |  |  | Benetutti |
| Baddu Lione | Strawberry tree ford | 181 III NE | E=512825  N=4517213 |  |  | Berchidda |
| Nodu de S’Olidone | Strawberry tree hill |  |  |  | X |  |
| Punta Solidone - *P. S’Olidone* | Strawberry tree peak | 181 II NO | E=522235  N=4512928 |  |  |  |
| Riu S’Olidone | Strawberry tree stream | 181 II SO | E=517857  N=4507912 |  |  |  |
| Scala Olidone | Steep climb of the strawberry tree | 181 II SO | E=518487  N=4507320 |  |  |  |
| Solidone - *S’Olidone* | The strawberry tree |  |  | X |  |  |
| Monte Selidone - *M. S’Elidone* | Strawberry tree mountain |  |  |  | X | Bessude |
| Riu Tuvulione - *Riu Tuvu Lione* | Stream of the strawberry tree gorge |  |  |  | X |  |
| Su Lidone | The strawberry tree |  |  | X |  |  |
| Tuvuglione- *Tuvu Lione* | Stream of the strawberry tree gorge | 193 IV SE | E=468947  N=4489298 |  |  |  |
| Su Lidone | The strawberry tree | 194 II NE | E=533981  N=4477898 |  |  | Bitti |
| Fonte S'Elidone | Strawberry tree spring | 193 II SE | E=491081  N=4471884 |  |  | Bolotana |
| Monte S'Elidone | Strawberry tree mountain | 193 II SE | E=491008  N=4471363 |  |  |  |
| Su Lidone | The strawberry tree |  | E=491286  N=4472157 | X |  |  |
| Tanca S' Elidone | Strawberry tree farm | 193 II SE | E=490721  N=4471914 |  |  |  |
| Schina su Lidone *- Ischina su Lidone* | Strawberry tree ridge | 194 III NO | E=498160  N=4477304 |  |  | Bono |
| Puttos de Lidone | Strawberry tree wells |  | E=494348  N=4479219 |  |  | Bonorva |
| S’ Elidone | The strawberry tree | 194 IV NO |  |  |  | Buddusò |
| Punta di lu Lione | Strawberry tree peak | 182 III SE | E=554875  N=4506503 |  |  | Budoni |
| Su Lidonarzu | The strawberry tree forest/maquis | 194 IV SE | E=509554  N=4483927 |  |  | Bultei |
| Bruncu Mitza de S’ Olioni | Rocky outcrop of the strawberry tree spring | 234 I NE | E=532150  N=4353303 |  |  | Burcei |
| Bruncu S’ Olioni | Rocky outcrop of the strawberry tree | 226 II SE | E=535069  N=4359400 |  |  |  |
| Monte Arcu S’ Olioni | Mountain of the strawberry tree arch | 226 II SE | E=537505  N=4356970 |  |  |  |
| Mitza de s’ Olioni | Strawberry tree spring | 234 I NE | E=532103  N=4353493 | X |  |  |
| Cungiau su Lidone | Fenced land of the strawberry tree |  |  | X |  | Busachi |
| Monte Lione | Strawberry tree mountain |  |  | X |  |  |
| Scala Olione | Steep climb of the strawberry tree |  |  |  | X |  |
| Strada Vicinale su Lidone | Strawberry tree road |  | E=493013  N=4427086 |  |  |  |
| Su Lidone | The strawberry tree |  | E=493052  N=4426531 |  |  |  |
| Su Lidone | The strawberry tree |  | E=491624  N=4429095 |  |  |  |
| Fontana lu Lioni | Strawberry tree spring | 181 I SE |  |  |  | Calangianus |
| Montigiu lu Lioni | Strawberry tree hill | 181 I NE |  |  |  |  |
| Riu Maccia lu Lioni | Stream of the strawberry tree maquis |  |  | X |  |  |
| Riu Lioni | Strawberry tree stream | 168 II SO |  |  |  |  |
| Scala di lu Lioni | Steep climb of the strawberry tree | 181 I SO | E=517217  N=4522573 |  |  |  |
| Stazzo Maccia di lu Lioni | Enclosure of the strawberry tree maquis | 181 I NO | E=522519  N=4530292 |  |  |  |
| Is Olionis | The strawberry trees (forest/maquis) | 233 II NE | E=494825  N=4332248 |  |  | Capoterra |
| Riu S’ Olioni | Strawberry tree stream |  | E=495417  N=4331101 | X |  |  |
| S’arcu de S’ Olioni | The strawberry tree arch | 233 II NE | E=495051  N=4331779 | X |  |  |
| S’Olioni | The strawberry tree |  |  | X |  |  |
| Cuccuru Planu de Olionis | Crown of the strawberry trees plain | 233 IV NO | E=461918  N=4344877 |  |  | Carbonia |
| Planu de Olionis | Strawberry trees plain | 233 IV NO | E=462620  N=4345115 |  |  |  |
| Riu S’Ega Olionis | Stream from the little valley of the strawberry trees |  | E=464201  N=4337010 |  |  |  |
| Monte Aliddoni | Strawberry tree mountain | 180 IV SE | E=473104  N=4526483 |  |  | Castelsardo |
| Casa Lidone | House of the strawberry tree | 180 II NE | E=491190  N=4515036 |  |  | Chiaramonti |
| Casa su Lidone | House of the strawberry tree | 180 II SE | E=490883  N=4509185 |  |  |  |
| Riu Monte Lidone | Stream from the strawberry tree mountain |  |  | X |  |  |
| Riu Su Lidone | Strawberry tree stream |  |  | X |  |  |
| Su Lidone | The strawberry tree |  | E=491354  N=4514981 |  |  |  |
| Bruncu S’Olioni | Rocky outcrop of the strawberry tree |  | E=516486  N=4361720 |  |  | Dolianova |
| Is Olionis | The strawberry trees |  |  | X |  |  |
| Monte Olioni | Strawberry tree mountain | 239 I NE | E=486605  N=4314030 |  |  | Domus de Maria |
| Pala Ollionis | Strawberry trees slope |  |  | X |  | Domusnovas |
| Pranus Olioni | Strawberry tree plain | 225 III SE | E=469658  N=4358415 | X |  |  |
| Punta Genna Olioni | Peak of the strawberry tree pass |  |  | X |  |  |
| Riu Planu S’Olioni | Stream of the strawberry tree plain |  |  | X |  |  |
| Serra Is Olionis di Edda | Ridge of the Edda’s strawberry trees | 225 III SE | E=467622  N=4362806 |  |  |  |
| Su de Is Olionis | The piece (of land) of the strawberry trees |  | E=469474  N=4354709 |  |  |  |
| Cuccuru is Olionis | Strawberry trees crown |  |  | X |  | Donori |
| Is Olionis | The strawberry trees | 226 III NE | E=513553  N=4364330 | X |  |  |
| Su Lidonargiu | The strawberry tree forest/maquis |  | E=537754  N=4472500 | X |  | Dorgali |
| Riu su Lidone | Strawberry tree stream |  | E=544860  N=4454695 |  |  |  |
| Su Lidone | The strawberry tree | 208 IV SO | E=544819  N=4454953 |  |  |  |
| Casa Lionaglia | House of the strawberry tree forest/maquis | 180 II NE | E=494285  N=4516300 |  |  | Erula |
| Casa Monte Lidone | House of the strawberry tree mountain |  | E=495459  N=4513787 |  |  |  |
| La Lionaglia | The strawberry tree forest/maquis |  | E=493843  N=4517263 |  |  |  |
| Su Lidonalzu | The strawberry tree forest/maquis |  | E=494154  N=4517504 |  |  |  |
| Is Coas ‘e Oglionaxiu | The extremity (of the land) of the strawberry tree forest/maquis | 226 I NE | E=529429  N=4389314 |  |  | Escalaplano |
| Riu S’Olionaxiu | Stream of the strawberry tree forest/maquis |  | E=533584  N=4390999 |  |  |  |
| Sa Matta S’Ollioni | The strawberry tree plant |  |  | X |  |  |
| Scala Olioni | Steep climb of the strawberry tree |  |  |  | X |  |
| Guardia de S’Utturu de S’Olioni | The lookout of the strawberry tree lane |  |  |  | X | Esterzili |
| Monte su Lidone | Strawberry tree mountain |  | E=469268  N=4497732 |  |  | Florinas |
| Enna de S’Olioni | Strawberry tree pass |  |  |  | X | Fluminimaggiore |
| Punta Manna Is Olionis | Big peak of the strawberry trees | 225 III NO | E=462951  N=4364388 |  |  |  |
| Punta S’Enna S’ Olioni | Peak of the strawberry tree pass |  |  |  | X |  |
| Scala Olioni | Steep climb of the strawberry tree |  |  |  | X | Fordongianus |
| Scala Sa Sedda S’Olioni | Steep climb of the strawberry tree valley |  |  |  | X |  |
| Bruncu S’Olioni | Rocky outcrop of the strawberry tree | 225 I SE | E=495876  N=4377584 |  |  | Furtei |
| Bruncu Perda Olioni | Rocky outcrop of the rocky place of the strawberry tree |  |  |  | X | Gadoni |
| Conca de S'Olioni | Strawberry tree hollow |  |  |  | X |  |
| Costa Campu de S'Olioni | Slope of the countryside of the strawberry tree |  |  |  | X |  |
| Perda (O)lioni | Rocky place of the strawberry tree |  |  | X |  |  |
| Genna Olioni/a | Strawberry tree pass |  |  |  | X | Gairo |
| Funtana su Lidone | Strawberry tree spring | 195 III SO | E=548747  N=4470096 |  |  | Galtellì |
| Scala de S’Olioni | Steep climb of the strawberry tree |  |  |  | X | Genoni |
| Su Bruncu ‘e S’Olioni | The rocky outcrop of the strawberry tree | 218 III NO | E=497635  N=4400785 |  |  | Gesturi |
| Scala(s) (de) (S’) Olioni(s) | Steep climb of the strawberry trees |  |  | X |  |  |
| Roja de Oglionis - *R. de Ollioni* | Strawberry tree stream |  |  |  | X | Gonnoscodina |
| Casa Genna S'Olioni | House of the strawberry tree pass |  | E=462341  N=4368233 |  |  | Gonnosfanadiga |
| Cuccuru Genna S’Olioni | Crown of the strawberry tree pass | 225 III NE | E=466355  N=4366989 |  |  |  |
| Genna S’Olioni | Strawberry tree pass | 225 III NO | E=462050  N=4368129 |  |  |  |
| Strada sa de S’Olioni | Strawberry tree road |  | E=462207  N=4367960 |  |  |  |
| Roja de Oglionis - *R. de Ollioni* | Strawberry tree stream |  |  |  | X | Gonnostramatza |
| Costa Ladolionis - *C. Ladu Olionis* | Slope of the wide stretch of the strawberry trees | 226 IV SO | E=504779  N=4381076 |  |  | Guamaggiore |
| Ladu de Olioni | Wide stretch of the strawberry tree |  |  | X |  |  |
| Cuccuru Perdilloni *- C. Perd’e Olioni* | Crown of the rocky place of the strawberry tree | 233 IV NO | E=457272  N=4347161 |  |  | Iglesias |
| Genna Olioni | Strawberry tree pass |  |  |  | X |  |
| Punta Genna Ollioni | Peak of the strawberry tree pass | 225 III SO | E=463015  N=4356632 |  |  |  |
| Sa Punta S’Olioni | The strawberry tree peak | 233 IV NO | E=454851  N=4351278 |  |  |  |
| Perda ‘e Lione | Rocky place of the strawberry tree | 219 IV SE | E=551676  N=4414784 |  |  | Ilbono |
| S’ Olioni / Su Lioni | The strawberry tree |  | E=550997  N=4413387 | X |  |  |
| Monte S' Elidone | Strawberry tree mountain |  | E=491780  N=4471408 |  |  | Illorai |
| Su Lidone | The strawberry tree |  |  | X |  | Irgoli |
| Casa Delidone - *C. De Lidone* | House of the strawberry tree |  | E=465366  N=4489473 |  |  | Ittiri |
| Casa su Lidonalzu | House of the strawberry tree forest/maquis |  | E=457457  N=4488921 |  |  |  |
| Delidone - *De Lidone* | (The Piece of land) of the strawberry tree |  |  |  | X |  |
| Funtana Puttos de Lidone | Spring of the strawberry tree wells | 193 IV SE | E=465315  N=4489553 |  |  |  |
| Su Lidonalzu | The strawberry tree forest/maquis | 193 IV SO | E=457312  N=4489234 |  |  |  |
| Puttos de Lidone | Strawberry tree wells | 193 IV SE | E=464912  N=4489936 |  |  |  |
| Monte Arbuticci | Strawberry tree mountain |  | E=539403  N=4564466 |  |  | La Maddalena |
| Prato S’Olioni | Strawberry tree field |  |  |  | X | Laconi |
| Rio Olione | Strawberry tree stream |  |  | X |  |  |
| S’Olione | The strawberry tree | 218 IV SO | E=504380  N=4413092 |  |  |  |
| Punta Puddelidone (?) - *Punta Puddone* (?) *de Lidone* | Peak of the shoot (?) of the strawberry tree |  |  |  | X | Laerru |
| Genna Olioni | Strawberry tree pass |  |  | X |  | Lanusei |
| Bruncu Lidonariu | Rocky outcrop of the strawberry tree forest/maquis |  |  |  | X | Loculi |
| Scala Lidone | Steep climb of the strawberry tree |  |  |  | X |  |
| Rio su Lidone | Strawberry tree stream |  |  | X |  | Lodè |
| Craru de su Lidone | Strawberry tree hill |  |  |  | X | Lodine |
| Rio su Lidonagliu | Stream of the strawberry tree forest/maquis |  |  | X |  | Lula |
| Scala Lidone | Steep climb of the strawberry tree |  |  |  | X |  |
| La Liunaglia | The strawberry tree forest/maquis |  | E=519327  N=4542094 |  |  | Luogosanto |
| Stazzo Lu Lioni | Strawberry tree enclosure | 168 II SO |  |  |  | Luras |
| Craru su Lidone | Strawberry tree hill | 207 I SO | E=519892  N=4451311 |  |  | Mamoiada |
| Fontana Lidone | Strawberry tree spring |  |  |  | X |  |
| Roja de Lidone | Strawberry tree stream |  |  |  | X |  |
| Su Lidone | The strawberry tree |  |  | X |  |  |
| Arcu S’Olioni | Strawberry tree arch | 234 I SE | E=537845  N=4342483 |  |  | Maracalagonis |
| Bruncu Olioni | Rocky outcrop of the strawberry tree |  |  | X |  |  |
| Guardia S’Olionargiu | The lookout of the strawberry tree forest/maquis |  |  |  | X |  |
| Perda de Sa Gora Su Lioneddu | Rocky place of the gorge of the small strawberry tree |  |  |  | X |  |
| Pranu Ollioni | Strawberry tree plain | 217 II NO | E=476703  N=4404418 | X |  | Marrubiu |
| Monte Lidone | Strawberry tree mountain | 180 II NO | E=483250  N=4516373 |  |  | Martis |
| Costa S’Olioni | Strawberry tree slope |  |  | X |  | Masullas |
| Bruncu Olione | Rocky outcrop of the strawberry tree |  | E=500208  N=4419473 |  |  | Meanasardo |
| Funtana Olione | Strawberry tree spring | 218 IV NO | E=501033  N=4419653 |  |  |  |
| Strada Funtana Olione | Road of the strawberry tree spring |  | E=501189  N=4420545 |  |  |  |
| Pala S’Olioni | Strawberry tree slope |  |  | X |  | Mogoro |
| Funtana Lidone / Ciuru Fontana Lidone | Strawberry tree spring/ ? strawberry tree spring | 207 III SO | E=502608  N=4434662 |  | X |  |
| Punta su Lidone | Strawberry tree peak |  |  |  | X |  |
| Bruncu S’Olioni | Rocky outcrop of the strawberry tree | 235 IV NO | E=549427  N=4352131 |  |  | Muravera |
| Giba S’Olioni | Strawberry tree hill | 235 IV NO | E=545671  N=4349329 |  |  |  |
| Conca S’Olioni | Strawberry tree hollow |  |  | X |  | Narcao |
| Costa Olioni | Strawberry tree slope | 233 IV SE | E=474043  N=4337442 |  |  |  |
| Punta de S’Olioni | Strawberry tree peak | 233 IV SE | E=472269  N=4340297 |  |  |  |
| S’arcu S’Olioni | The strawberry tree arch |  |  | X |  |  |
| S’Ega Orionis | The little valley of the strawberry trees |  | E=467962  N=4341126 | X |  |  |
| Serra Is Orionis | Strawberry trees ridge | 233 IV SE | E=464557  N=4341134 |  |  |  |
| Lidones | The strawberry trees |  | E=503817  N=4489112 |  |  | Nughedu San Nicolò |
| Su Lidone | The strawberry tree |  |  | X | X | Nule |
| Vachile de Su Lidoni | Strawberry tree cowbam |  |  |  | X |  |
| Funtana ‘e Lidone | Strawberry tree spring | 207 I NE | E=533651  N=4464120 |  |  | Nuoro |
| Riu Funtana Lidone | Stream from the strawberry tree spring |  | E=533960  N=4464585 |  |  |  |
| Riu su Lidone | Strawberry tree stream | 207 I NO | E=519298  N=4464107 |  |  |  |
| Su Lidone | The strawberry tree |  |  | X |  |  |
| Mitza S’ Olioni | Strawberry tree spring | 218 III NO | E=501177  N=4401476 |  |  | Nuragus |
| Scala S’ Olioni | Steep climb of the strawberry tree | 218 III NO | E=500922  N=4401267 |  |  |  |
| Funtana Olioni | Strawberry tree spring |  |  | X |  | Nurri |
| Is Olionis | The strawberry trees | 233 I SO | E=478214  N=4336683 |  |  | Nuxis |
| Is Orionis | The strawberry trees |  | E=476889  N=4335661 |  |  |  |
| S’arcu de S’Olioni | The strawberry tree arch | 233 II NO | E=483548  N=4330918 |  |  |  |
| Punta Leoneddu | Peak of the small strawberry tree |  | E=561499  N=4524458 |  |  | Olbia |
| Cuili su Lidone | Strawberry tree sheepfold | 208 IV SO | E=539366  N=4454695 |  |  | Oliena |
| Crastu de S’Olidone | Rocky place of the strawberry tree |  |  |  | X |  |
| Funtana su Lidone | Strawberry tree spring | 195 III SO | E=539109  N=4467120 |  |  |  |
| Riu su Lidone | Strawberry tree stream | 194 II SE | E=538073  N=4467915 |  |  |  |
| Serra Su Lidone | Strawberry tree ridge | 208 IV SO | E=539426  N=4453287 |  |  |  |
| Su Lidone / Solidone - *S’Olidone* | The strawberry tree | 194 II SE | E=538561  N=4467454 |  | X |  |
| Su Lidone | The strawberry tree | 207 III NE | E=509775  N=4444969 |  |  | Olzai |
| Nuraghe Tuppa Lidone | Nuraghe of the strawberry tree maquis | 194 III SE | E=512738  N=4467796 |  |  | Oniferi |
| Craru de su Lidone | Strawberry tree hill |  |  |  | X | Orani |
| Su Lidone | The strawberry tree |  |  | X |  |  |
| Gutturu su Lidone | Strawberry tree lane | 207 II NE | E=533257  N=4449211 |  |  | Orgosolo |
| Gutturu su Lidone | Strawberry tree lane | 207 I SE | E=536571  N=4440626 |  |  |  |
| Genna Olioni | Strawberry tree pass |  |  |  | X | Oridda (hamlet of Villacidro) |
| Ollionis | The strawberry trees |  |  | X | X | Oristano |
| Su Lidonargiu | The strawberry tree forest/maquis | 195 III SE | E=555474  N=4469588 | X |  | Orosei |
| Sulidone - *Su Lidone* | The strawberry tree | 195 II NO | E=564955  N=4477607 |  |  |  |
| Is Olionis | The strawberry trees | 226 I NO | E=519990  N=4389433 |  |  | Orroli |
| Sa Pala de is Ollionis | Strawberry tree slope |  |  | X |  |  |
| Fontana Lidone | Strawberry tree spring |  |  |  | X | Ortueri |
| Su Lidone | The strawberry tree | 194 II SE | E=535178  N=4472839 |  |  | Orune |
| Case lu Lione Toltu | Houses of the crooked strawberry tree | 181 III NO | E=505193  N=4519520 |  |  | Oschiri |
| S’ Olidone | The strawberry tree |  |  |  | X |  |
| Monte Lidone | Strawberry tree mountain | 180 IV SE |  |  |  | Osilo |
| Su Lioni | The strawberry tree |  |  | X |  | Osini |
| Rio Terra di Lione | Stream of the strawberry tree land |  |  | X |  | Ovodda |
| S’ Elidone | The strawberry tree |  | E=540266  N=4501270 |  |  | Padru |
| Pinnetta Elidone | Strawberry tree hut | 194 IV SE | E=513581  N=4490592 |  |  | Pattada |
| Riu Elidone - *R. ‘e Lidone* | Strawberry tree stream |  |  | X |  |  |
| Costa de Su Lioni | Strawberry tree slope |  |  |  | X | Pauli Arbarei |
| Casa Tupulidone - *C.* *Tupa ‘e Lidone* | House of the strawberry tree maquis |  | E=483610  N=4432106 |  |  | Paulilatino |
| Tupulidone - *Tupa ‘e Lidone* | Strawberry tree maquis | 206 II SO | E=483411  N=4432045 |  |  |  |
| Se Lioni *-* *S’Elioni* | The strawberry tree |  |  | X |  | Perdasdefogu |
| Sedda de Lioni | Strawberry tree valley | 227 IV NO | E=539106  N=4389422 |  |  |  |
| Case su Lione | Houses of the strawberry tree | 181 III NO | E=498041  N=4518806 |  |  | Perfugas |
| Funtana Monte lidone | Spring from the strawberry tree mountain | 180 II NE | E=493325  N=4518077 |  |  |  |
| Riu Ena di Lioni | Stream from the strawberry tree spring |  | E=498879  N=4518935 |  |  |  |
| Solidonalzu *- S’ Olidonalzu* | Strawberry tree forest/maquis | 180 II NE |  |  |  |  |
| Trainu de Lione | Strawberry tree stream |  |  |  | X | Ploaghe |
| Li Lioni | The strawberry trees | 179 II NE | E=452532  N=4517287 |  |  | Porto Torres |
| Riu Tanca di li Lioni | Stream of the strawberry tree farm |  |  | X |  |  |
| Fontana Lioni | Strawberry tree spring |  |  |  | X | Posada |
| Punta Lione | Strawberry tree peak |  |  |  | X |  |
| Guardia S’Olioni / Guardi de Pranu de S’Olioni | The lookout of the strawberry tree/ The lookout of the strawberry tree plateau |  |  | X | X | Pula |
| Mori S’Arcu de S’Olioni | Lane of the strawberry tree arch |  |  |  | X |  |
| Punta S’Olionargiu | Peak of the strawberry tree forest/maquis |  | E=487787  N=4318496 |  |  |  |
| Punta S’Olioni | Strawberry tree peak | 233 II SE | E=491118  N=4318927 |  |  |  |
| Puntixedda S’Olioni | Little peak of the strawberry tree |  |  | X |  |  |
| S’Arcu S’Olioni | The strawberry tree arch | 233 II SE | E=490914  N=4319075 |  |  |  |
| S’ Olioni | The strawberry tree | 233 II SE | E=493576  N=4322031 |  |  |  |
| Riu su Lidonazu | Stream of the strawberry tree forest/maquis | 193 IV SO | E=457461  N=4490190 |  |  | Putifigari |
| Conca S’Olioni | Strawberry tree hollow |  |  | X |  | Riola Sardo |
| Is Olionis | The strawberry trees | 206 III SO | E=459682  N=4429607 | X |  |  |
| Pischina S’Olioni | Strawberry tree swamp |  |  | X |  |  |
| Rio Olioni | Strawberry tree stream |  |  |  | X | Ruinas |
| Funtana Lioni | Strawberry tree spring | 218 II NO | E=521706  N=4403141 |  |  | Sadali |
| Guardia de S’Utturu de S’Olioni | The lookout of the strawberry tree lane |  |  |  | X |  |
| Perda S’Olioni | Rocky place of the strawberry tree |  |  |  | X |  |
| S’Olioni | The strawberry tree |  |  | X |  | Samatzai |
| Genna Olioni | Strawberry tree pass |  |  | X |  | San Basilio |
| Benatzu Su de Lionis | Swampy area of the strawberry tree | 233 III NO |  |  |  | San Giovanni Suergiu |
| Is Lionis | The strawberry trees | 233 III NO | E=460262  N=4328709 |  |  |  |
| Su de Lionis | The piece (of land) of the strawberry trees |  | E=460085  N=4328945 |  |  |  |
| Su Medau de Lionis | The sheepfold of the strawberry trees |  |  | X |  |  |
| Su Prano de S’Orioni | The plateau of the strawberry tree |  | E=456214  N=4332569 | X |  | San Giovanni Suergiu |
| Bruncu su Lioni | Rocky outcrop of the strawberry tree | 226 II NO | E=525346  N=4365467 |  |  | San Nicolò Gerrei |
| Cuccuru Lioni Fois / Cuccuru Olioni today known as S’Olionaxeddu | Strawberry tree crown that belongs to Mr. Fois today known as small strawberry tree forest/maquis |  | E=528397  N=4369043 | X |  |  |
| Via Lioni | Strawberry tree road |  |  |  | X |  |
| S’Ollione | The strawberry tree | 226 I SE | E=529435  N=4375373 | X |  |  |
| Lu Lioni | The strawberry tree | 182 III NE | E=553457  N=4514763 |  |  | San Teodoro |
| Punta S’ Olioni | Strawberry tree peak | 227 II NO | E=542209  N=4364393 |  |  | San Vito |
| Serra S’ Olioni | Strawberry tree ridge | 227 II SO | E=540868  N=4357285 |  |  |  |
| Monte Lionis | Strawberry trees mountain | 225 I SE | E=490994  N=4380527 |  |  | Sanluri |
| Sedda S’Olioni | Strawberry tree valley |  | E=521107  N=4368677 |  |  | Sant’Andrea Frius |
| Cammino Oglionis - *Cammino Ollionis* | Strawberry tree lane |  |  |  | X | Santa Giusta |
| Arcu S’Olioni | Strawberry tree arch | 233 II SO | E=482567  N=4323317 |  |  | Santadi |
| Punta de S’Olione | Strawberry tree peak |  |  |  | X |  |
| Montigiu lu Lioni | Strawberry tree hill |  | E=531943  N=4538792 |  |  | Sant'Antonio di Gallura |
| Riu Lioni | Strawberry tree stream |  | E=526414  N=4540281 |  |  |  |
| Stazzo Lu Lioni | Strawberry tree enclosure |  | E=523482  N=4539143 |  |  |  |
| Craru de su Lidone | Strawberry tree hill |  |  |  | X | Sarule |
| Su Lidoni | The strawberry tree |  |  | X |  |  |
| Casa S’ Alidoni | House of the strawberry tree | 179 II SE | E=443276  N=4502739 |  |  | Sassari |
| Riu S' Alidoni | Strawberry tree stream |  | E=443531  N=4504297 |  |  |  |
| S’ Alidoni | The strawberry tree | 179 II SE |  |  |  |  |
| Serra di Lioni | Strawberry tree ridge | 180 III SO | E=457167  N=4510155 |  |  |  |
| Nuraghe de S’Olioni | Nuraghe of the strawberry tree |  |  |  | X | Sedini |
| Monte Ollioni | Strawberry tree mountain | 234 IV SE | E=499457  N=4378977 |  |  | Segariu |
| Su Lidone | The strawberry tree | 206 III SE | E=467048  N=4434381 |  |  | Seneghe |
| Is Ollionis | The strawberry trees |  |  | X |  | Senis |
| Genna Olioni | Strawberry tree pass | 226 IV SE | E=512761  N=4375879 | X |  | Arixi (hamlet of Senorbì) |
| S’arcu S’Olioni | The arch of the strawberry tree |  |  | X |  | Serbariu (hamlet of Carbonia) |
| S’Ega is (S’) Olioni(s) | The little valley of the strawberry trees |  |  | X |  |  |
| Bruncu S’Olioni | Rocky outcrop of the strawberry tree |  | E=517600  N=4363169 | X |  | Serdiana |
| (S’) Olioni | (The) strawberry tree |  |  |  | X |  |
| Costa S’Ollioni | Strawberry tree slope | 226 IV SO | E=500710  N=4373569 |  |  | Serrenti |
| Mitza de S’Olioni | Strawberry tree spring |  |  |  | X | Settimo san Pietro |
| Baccu Lioni | Strawberry tree gorge | 217 II SE | E=495040  N=4397805 |  |  | Setzu |
| Strada Vicinale Baccu Lioni | Road of the strawberry tree gorge |  | E=495515  N=4398875 |  |  |  |
| Funtana su Lioni | Strawberry tree spring | 218 II NO | E=525617  N=4407799 |  |  | Seui |
| Riu de Genna Liones | Stream from the strawberry trees pass |  |  |  | X |  |
| Vau de Genna Liones | Ford of the strawberry trees pass |  |  |  | X |  |
| Bau Gena Liones - *B. Genna* *Liones* | Ford of the strawberry trees pass |  |  |  | X | Seulo |
| Genna Lioni | Strawberry tree pass | 218 II SO | E=523547  N=4413092 |  |  |  |
| Riu Genaliones - *R. Genna Liones* | Stream of the strawberry tree pass |  |  |  | X |  |
| Strada Comunale Genna Lioni | Local road of the strawberry tree pass |  | E=523505  N=4413318 |  |  |  |
| Bruncu Olioni | Rocky outcrop of the strawberry tree |  |  |  | X | Siamanna |
| Scala s’Olioni | Steep climb of the strawberry tree | 217 I NE | E=481874  N=4418578 |  |  | Siapiccia |
| Is Olionis | The strawberry trees |  | E=481291  N=4419846 |  |  |  |
| Punta Seddas Is Olionis | Peak of the strawberry trees valley |  | E=486446  N=4336651 | X |  | Siliqua |
| Sa Sedda Is Olionis | The strawberry trees valley |  | E=486255  N=4336414 |  |  |  |
| Bruncu Elioni | Rocky outcrop of the strawberry tree |  |  |  | X | Simaxis |
| Olidoni | Strawberry tree | 217 II NE | E=491480  N=4400864 |  |  | Sini |
| Fruncu S' Ulidone | Strawberry tree plateau | 195 IV NE | E=557073  N=4494733 |  |  | Siniscola |
| Nodu Mannu de Su Lidonargieddu | Big hill of the small strawberry tree forest/maquis |  |  |  | X |  |
| Punta Lidone | Strawberry tree peak |  |  |  | X |  |
| Riu su Lidone | Strawberry tree stream | 195 II NO |  |  |  |  |
| Su Lidone | The strawberry tree | 195 II NO | E=566864  N=4482159 |  |  |  |
| S’Ulidone - *Su Lidone* | The strawberry tree | 195 IV SE | E=556560  N=4484021 |  |  |  |
| Arcu de S’ Olioni | Arch of the strawberry tree |  |  |  | X | Sinnai |
| Bruncu S’ Olioni | Rocky outcrop of the strawberry tree | 234 I SE | E=537831  N=4342815 |  |  |  |
| Olioni | Strawberry tree |  |  |  | X |  |
| Rocca s’Olioni | Strawberry tree boulder |  |  |  | X |  |
| Genna Olioni | Strawberry tree pass |  | E=518948  N=4378042 | X |  | Siurgus Donigala |
| Canale S’Olioni | Strawberry tree hollow |  |  | X |  | Soleminis |
| Mitza S’ Olioni | Strawberry tree spring | 226 III SE | E=515368  N=4353980 |  |  |  |
| Rio Mitza s’Olioni | Stream from the strawberry tree spring |  |  | X |  |  |
| S’Ollioni | The strawberry tree |  |  | X |  |  |
| Conca de S’Olioni | Strawberry tree hollow |  |  |  | X | Sorgono |
| Fontana Lioni | Strawberry tree spring |  |  |  | X |  |
| S’Oliones | The strawberry trees |  |  | X |  |  |
| Cuile su Lione | Strawberry tree sheepfold | 208 III SE | E=549373  N=4430071 |  |  | Talana |
| Rio Olione | Strawberry tree stream |  |  | X |  |  |
| S’Olione | The strawberry tree |  |  | X |  |  |
| Su Monte su Lionargiu | The mountain of the strawberry tree forest/maquis |  | E=541356  N=4431114 |  |  |  |
| Fontana lu Lioni | Strawberry tree spring |  | E=529286  N=4528758 |  |  | Telti |
| Canale Olioni | Strawberry tree hollow | 168 II NO | E=518577  N=4552702 |  |  | Tempio Pausania |
| Conca de l’Olioni / Conca de su Leone | Strawberry tree hollow | 182 IV SO |  |  |  |  |
| Punta di Lu Lione | Strawberry tree peak |  |  |  | X |  |
| Rio lu Lioni | Strawberry tree stream | 182 IV SE |  |  |  |  |
| Scala di Lilioni - *S. di li Lioni* | Steep climb of the strawberry tree | 181 IV SE | E=509876  N=4522467 |  |  |  |
| Stazzi lu Lioni | Strawberry tree enclosures | 168 III SE |  |  |  |  |
| Coa de Lione | The extremity (of the land) of the strawberry tree |  | E=475081  N=4521172 |  |  | Tergu |
| Monte Lidone | Strawberry tree mountain |  | E=474446  N=4521974 |  |  |  |
| Cuile Lionis | Strawberry trees sheepfold | 219 III SE | E=556620  N=4395376 |  |  | Tertenia |
| Foxi Lioni | Strawberry tree river outlet | 227 IV NE | E=555808  N=4390023 |  |  |  |
| Rio Foxi Lioni | Strawberry tree river outlet |  |  | X |  |  |
| Riu Perd’e Lioni | Stream of the rocky place of the strawberry tree |  | E=555214  N=4390395 |  |  |  |
| Bruncu de su Elidone | Rocky outcrop of the strawberry tree |  |  |  | X | Teti |
| Craru Lidone | Strawberry tree hill | 207 III NE | E=509366  N=4443527 |  |  |  |
| Canale de S’Olioni | Strawberry tree hollow | 233 II SO | E=478933  N=4318186 |  |  | Teulada |
| Cuccuru S’Olioni | Strawberry tree crown | 233 II SO | E=477338  N=4320341 |  |  |  |
| Riu S’Olioni | Strawberry tree stream | 233 III SE | E=471658  N=4317530 |  |  |  |
| Fontana Lidone | Strawberry tree spring |  |  |  | X | Tiana |
| Erillione *- Riu Illione* | Strawberry tree stream | 207 III SE | E=512113  N=4428595 |  |  | Tonara |
| Ianna Lioni | Strawberry tree pass | 182 III SE | E=550124  N=4505672 |  |  | Torpè |
| S’Ulidone | The strawberry tree | 195 IV NE | E=554248  N=4494927 |  |  |  |
| Case Foxilioni - *C. Foxi Lioni* | Houses of the strawberry tree river outlet |  | E=557553  N=4415723 |  |  | Tortolì |
| Foxiglioni - *Foxi Lioni* | Strawberry tree river outlet | 219 IV SE | E=558149  N=4415852 |  |  |  |
| Riu Foxiglioni - *R. Foxi Lioni* | Strawberry tree river outlet | 219 IV SE | E=557607  N=4415664 |  |  |  |
| Nuraghe Genna Olidone | Nuraghe of the strawberry tree pass |  | E=554969  N=4428701 |  |  | Triei |
| Costa de su Lioni | Strawberry tree slope |  |  |  | X | Tuili |
| Baccu Lioni | Strawberry tree gorge |  | E=545334  N=4390341 | X |  | Ulassai |
| Rio Baccu Lioni / Rio Olioni | Stream of the strawberry tree gorge/ Strawberry tree stream | 227 IV NO |  | X |  |  |
| Serra su Lioni | Strawberry tree ridge |  |  | X |  |  |
| Punta Olidone | Strawberry tree peak | 208 III SO | E=546231  N=4435082 |  |  | Urzulei |
| Riu S’Olidone | Strawberry tree stream | 208 IV SO | E=544755  N=4451052 |  |  |  |
| Strada Comunale Figaizzu S' Olioni | Local road of the strawberry tree figaizzu ? |  | E=484929  N=4408564 |  |  | Usellus |
| Roja de Oglionis - *R. de Ollioni* | Stream of the strawberry tree |  |  |  | X | Escovedu (hamlet of Usellus) |
| Bruncu Lionis | Rocky outcrop of the strawberry tree | 217 II SE | E=490587  N=4394626 |  |  | Ussaramanna |
| Pizzu Lioni | Strawberry tree top | 218 I SE | E=536484  N=4413802 |  |  | Ussassai |
| Selioni - *S’Elioni* | The strawberry tree |  |  | X |  |  |
| Serra Lioni | Strawberry tree ridge |  | E=535998  N=4412562 | X |  |  |
| Su Lionargiu | The strawberry tree forest/maquis |  | E=536656  N=4410865 |  |  |  |
| Su Pranu Is Olionis | The plain of the strawberry trees |  |  | X |  | Uta |
| L’Ulione | The strawberry tree |  |  | X |  | Valledoria |
| Cuccuru Olionis / Su Cuccuru de S’Olioni | Strawberry trees crown/ The crown of the strawberry tree |  | E=478759  N=4356977 |  |  | Vallermosa |
| Punta S’Olioni | Strawberry tree peak |  |  | X |  | Villa San Pietro |
| Puntixeda S’Olioni | Little peak of the strawberry tree |  |  | X |  |  |
| Rio S’Olioni | Strawberry tree stream |  |  |  | X |  |
| Strada sa Mitza e s' Olioni | Road of the strawberry tree spring |  | E=490723  N=4413885 |  |  | Villa Sant’Antonio |
| Monte Olioni | Strawberry tree mountain | 225 II SO | E=482475  N=4362794 |  |  | Villacidro |
| Pala Ollionis di Edda | Slope of Edda’s strawberry trees | 225 III SE | E=467050  N=4363317 | X |  |  |
| Pixina Ollioni | Strawberry tree swamp |  | E=484463  N=4370167 | X |  |  |
| Punta Olionis | Strawberry trees peak |  |  |  | X |  |
| Accu su Lione | Strawberry tree gorge |  | E=532619  N=4426125 |  |  | Villagrande Strisali |
| Arcu su Lione | Strawberry tree arch | 218 I NE | E=532111  N=4426855 |  |  |  |
| Bruncu de su Lione / Bruncu Elione - *B: ‘e Lione* | Rocky outcrop of the strawberry tree |  |  |  | X |  |
| Conca su Lidone | Strawberry tree hollow |  |  |  | X |  |
| Perda de su Lione | Rocky place of the strawberry tree |  |  |  | X |  |
| Punta de su Lidone | Strawberry tree peak |  |  |  | X |  |
| Rio Accu Lioni | Stream of the strawberry tree gorge |  |  | X |  |  |
| Sedda su Lione | Strawberry tree valley |  |  |  | X |  |
| Serra Lidone | Strawberry tree ridge |  |  |  | X |  |
| Su Lidone | The strawberry tree | 207 II SE | E=531329  N=4430950 |  |  |  |
| Baccu Olioni | Strawberry tree gorge |  |  | X |  | Villamar |
| Ollioni | Strawberry trees |  | E=498089  N=4387176 |  |  |  |
| Riu S’Ollioni | Strawberry tree stream | 226 IV NO | E=498259  N=4387413 |  |  |  |
| Acqua Solioni - *A. S’Olioni* | Strawberry tree water |  |  |  | X | Villamassargia |
| Planu de Olionis | Plain of the strawberry trees | 233 IV NE | E=466052  N=4345131 |  |  |  |
| Punta Sega S’Olioni – *Punta s’ega s’Olioni* | Peak of the little valley of the strawberry tree |  |  |  | X |  |
| Riu Gutturu di Mannolioni- *R. G. di Mannu Lioni* | Stream of the lane of the big strawberry tree | 233 IV SE | E=472203  N=4341859 | X |  |  |
| S’Arcu S’Olioni | The arch of the strawberry tree | 233 I SO | E=474525  N=4341874 |  |  |  |
| Serra su Lioni | Strawberry tree ridge |  | E=517923  N=4407008 |  |  | Villanovatulo |
| Baccu Olione | Strawberry tree gorge | 227 IV SE | E=545244  N=4375461 |  |  | Villaputzu |
| Baccu Olioneddu | Gorge of the small strawberry tree |  | E=544254  N=4375664 |  |  |  |
| Bruncu S’Olione | Rocky outcrop of the strawberry tree |  | E=545459  N=4375169 |  |  |  |
| Cuile S’Olione | Strawberry tree sheepfold | 227 IV SE | E=553113  N=4377930 |  |  |  |
| Bruncu s’Olioni | Rocky outcrop of the strawberry tree |  |  |  | X | Villasalto |
| Mitza S'Olioni | Strawberry tree spring |  | E=540588  N=4368799 |  |  |  |
| Bruncu Olioni | Rocky outcrop of the strawberry tree |  |  |  | X | Zepara (hamlet of Ales) |

IGM^a^ = Phyto-toponyms in IGM (Italian Military Geografic Institute) maps for which the respective IGM tablet number have been given.

WS^b^ = Phyto-toponyms available on the web site of the Autonomous Region of Sardinia for which the respective coordinates (WGS84/UTM zone 32 N) have been given.

CM^c^ = Phyto-toponyms in cadastral maps.

SA^d^ = Phyto-toponyms in the State Archive in Cagliari.
