# Supplementary material for: Phyto-toponyms of Arbutus unedo L. and their distribution in Sardinia (Italy)
Source: PLoS One. 2017 Jul 13;12(7):e0181174. doi: 10.1371/journal.pone.0181174 (PMC5509287; doi:10.1371/journal.pone.0181174)
Supplement: S2 Table — (DOC) [file pone.0181174.s002.doc]

**S2 Table. Place names not related to the strawberry tree in Sardinia but which, due to similar sound, seem to refer to the species.**

| **Place names in Sardinian language** | **Meaning** | **Sources consulted** | | | | **Municipality** |
| --- | --- | --- | --- | --- | --- | --- |
|  |  | **IGM^a^** | **WS^b^** | **CM^c^** | **SA^d^** |  |
| Punta del Leone | Peak of the lion | 192 IV NE | E=428077  N=4497323 |  |  | Alghero |
| Cuile Lianeddu | Anthroponym:  Julian's sheepfold | 227 IV SO | E=539235  N=4376686 |  |  | Armungia |
| Lianeddu | Anthroponym:  Julian | 227 IV SO | E=539041  N=4376830 |  |  |  |
| Rio Lianeddu | Anthroponym:  Julian's stream |  | E=539373  N=4376787 | X |  |  |
| Conc’Ailloni - *Conc’e Pilloni* | Bird head | 217 IV NO |  |  |  | Cabras |
| Nuraghe Conc’Ailloni - *N. Conc’e Pilloni* | Nuraghe of the bird head | 217 IV NO |  |  |  |  |
| Punta Conc’Ailloni - *Punta Conc’e Pilloni* | Peak of the bird head | 217 IV NO |  |  |  |  |
| Casa Leone | Anthroponym:  The Leone's (Sardinian surname) House |  | E=440338  N=4336809 |  |  | Carloforte |
| Punta Leone | Peak of Leone (Sardinian surname) | 232 II NO | E=439932  N=4333445 |  |  |  |
| Olion(n)as | Genus *Olea* | 206 III NO | E=457100  N=4441155 |  |  | Cuglieri |
| Casa Fortelioni - *Casa Forteleoni* | Anthroponym:  The Fortelioni (Sardinian surname) House |  | E=501898  N=4434300 |  |  | Neoneli |
| Funtana Leone | Anthroponym:  Leone's (Sardinian surname) Spring | 207 IV SO | E=497803  N=4453487 |  |  | Noragugume |
| Pedraglione - *Su Pedralzòne* | Rocky place |  | E=562000  N=4524639 |  |  | Olbia |
| S' Alisone | Genus *Alnus* |  | E=541023  N=4513909 |  |  | Padru |
| Riu Antoni Lioni | Anthroponym:  Anthony Lioni's stream |  | E=534721  N=4389149 | X |  | Perdasdefogu |
| Bilione | Anthroponym:  Bilione |  | E=476700  N=4506332 |  |  | Ploaghe |
| Cantoniera Bilione | Anthroponym:  The Bilione's road inspector's house | 180 II SO | E=476723  N=4505706 |  |  |  |
| Funtana Bilione | Anthroponym:  Bilione's spring | 180 II SO | E=476644  N=4507100 |  |  |  |
| Noraghe Bilione | Anthroponym:  Bilione's nuraghe |  | E=476332  N=4506928 |  |  |  |
| Rio Bilione | Anthroponym:  Bilione's stream |  |  |  | X |  |
| Stazzo Bulioni - *Stazzo Ulioni* | Enclosure of the whirlwind |  | E=529871  N=4539302 |  |  | Sant’Antonio di Gallura |
| Alisones | Genus *Alnus* | 206 I SO | E=476274  N=4448789 |  |  | Santu Lussurgiu |
| Funtana Alisones | Genus *Alnus* | 206 I SO | E=476498  N=4448413 |  |  |  |
| Santu Lioni | Saint Lion |  |  | X |  | Tuili |
| Serra de Saiglionis- *Serra de S’Aionis* | Ridge of the garlic |  | E=540115  N=4401310 | X |  | Ulassai |
| Strada Vicinale de Serra Saiglionis *- Strada vicinale de Serra S’Aionis* | Road of the ridge of the Garlic |  | E=539380  N=4401162 |  |  |  |
| Su Saltu de Monteleoneddu - *Su Saltu de Monte Leoneddu* | Villanova Monteleone countryside |  | E=462516  N=4487222 |  |  | Villanova Monteleone |
| Trainu de Leone | Villanova Monteleone stream | 193 IV SO |  |  |  |  |
| Monte Padrillonis *- Monte Padru de Agrillonis* | Mountain of the asphodel field | 217 I SE |  |  |  | Villa Sant’Antonio |
| Padrillonis - *Agrillonis* | Asphodel field |  |  | X |  |  |
| Strada vicinale Pardillonis - *Strada vicinale Padru de Agrillonis* | Road of the asphodel field |  |  | X |  |  |
| Salto di Gemilioni | Slope of Gemilioni  (Gemilioni is a personal performance during the Judicial period in Sardinia) |  |  |  | X | Villaspeciosa |

IGM^a^ = Place names in IGM (Italian Military Geografic Institute) maps for which the respective IGM tablet number have been given.

WS^b^ = Place names available on the web site of the Autonomous Region of Sardinia for which the respective coordinates (WGS84/UTM zone 32 N) have been given.

CM^c^ = Place names in cadastral maps.

SA^d^ = Place names in the State Archive in Cagliari.
