# Supplementary material for: Phyto-toponyms of Arbutus unedo L. and their distribution in Sardinia (Italy)
Source: PLoS One. 2017 Jul 13;12(7):e0181174. doi: 10.1371/journal.pone.0181174 (PMC5509287; doi:10.1371/journal.pone.0181174)
Supplement: S3 Table — (DOC) [file pone.0181174.s003.doc]

**S3 Table. Place names of uncertain meaning but which, due to similar sound, seem to refer to the strawberry tree in Sardinia.**

| **Place names in Sardinian language** | **Meaning** | **Sources consulted** | | | | **Municipality** |
| --- | --- | --- | --- | --- | --- | --- |
|  |  | **IGM^a^** | **WS^b^** | **CM^c^** | **SA^d^** |  |
| Marina di Lioneddu | Shore of the small strawberry tree ? | 192 IV NE | E=428908  N=4497793 |  |  | Alghero |
| Miniera S’Arcillonis | Mine of the strawberry tree arch ? |  |  | X |  | Burcei |
| Terra ‘e Lisone | Land of the strawberry tree ? | 206 II SE | E=490000  N=4431105 |  |  | Busachi |
| Funtana dell’Azzo | Spring of the strawberry tree flower ? |  | E=516555  N=4524823 |  |  | Calangianus |
| Monte Lisone Ovest | Strawberry tree western mountain ? |  | E=463859  N=4364600 |  |  | Fluminimaggiore |
| Perdaliona | Rocky place of the strawberry tree ? |  |  | X |  | Gairo |
| Monte Lisone | Strawberry tree mountain ? |  | E=464898  N=4364757 |  |  | Gonnosfanadiga |
| Punta Olionica | Peak of the strawberry tree forest/maquis ? |  |  | X |  | Iglesias |
| Monte Mastru Lioni | Mountain of the mainmast Strawberry tree ?  Surname ? |  | E=516243  N=4545407 |  |  | Luogosanto |
| Nuraghe Puncilioni | Strawberry tree ? nuraghe |  | E=551273  N=4361787 |  |  | Muravera |
| Monteleone | Villanova Monteleone village? |  |  | X |  | Noragugume |
| Casa de s’Azzo’ | House of the strawberry tree flower ? |  | E=541143  N=4523023 |  |  | Olbia |
| Serra Lisone | Strawberry tree ridge ? |  | E=507559  N=4504104 |  |  | Oschiri |
| Casa Orioni | Strawberry tree ? house |  | E=466111  N=4333127 |  |  | Perdaxius |
| Via de Lione faj | Road of the strawberry tree ? |  |  |  | X | San Nicolò Gerrei |
| Olionara | Strawberry tree forest/maquis ? |  |  | X |  | Scano di Montiferro |
| Lionisa | Strawberry tree forest/maquis ? |  | E=496135  N=4448007 |  |  | Sedilo |
| Iscia Lioni | Vegetable garden of the strawberry tree ? |  |  |  | X | Sennori |
| Perdulioni | Rocky place of the strawberry tree ? |  |  | X |  | Seui |
| Punta Olionica | Peak of the strawberry tree forest/maquis ? |  |  | X |  | Siliqua |
| Bruncu Bioni - *Bruncu Obioni* | Rocky outcrop of the strawberry tree ? |  |  |  | X | Simaxis |
| Riu Gurdulionis or  Riu Gurdulonis | Stream of the strawberry tree ? | 226 I NO | E=523693  N=4384159 |  |  | Siurgus Donigala |
| Iscia Lioni | Vegetable garden of the strawberry tree ? |  |  |  | X | Sorso |
| Bolta de Leone - *Borta de Leone* | Place surrounded by strawberry tree ? | 193 IV SE | E=468924  N=4489295 |  |  | Thiesi |
| L’Aglioni | The strawberry tree ? | 180 I NE | E=493490  N=4535117 |  |  | Trinità d’Agultu e Vignola |
| Calioniga | The strawberry tree forest/maquis ? |  | E=497736  N=4394652 |  |  | Tuili |
| Serra Aglionis - *Serra A Lionis* | Strawberry tree ridge ? |  | E=536837  N=4397171 |  |  | Ulassai |
| Su Cuccuru de Su Obioni | Strawberry tree crown ? |  | E=480100  N=4356061 |  |  | Vallermosa |
| Trattalionis | ? of the strawberry trees |  | E=497831  N=4383323 |  |  | Villamar |
| Arco Saglione - *Arcu S’Olione* | Strawberry tree arch ? |  |  |  | X | Villamassargia |
| Casa di Mannolioni - *Casa di Mannu Lioni* | House of the big strawberry tree ? |  | E=4721123  N=4342073 |  |  |  |
| Mannalione *- Mannu Lione* | Big strawberry tree ? |  |  | X |  |  |
| Riu Surreglione - *Riu Su Lione* | Stream of the strawberry tree ? |  | E=545852  N=4379408 |  |  | Villaputzu |
| Meli Oi | Strawberry tree fruit ? |  |  | X |  | Villasalto |

IGM^a^ = Phyto-toponyms in IGM (Italian Military Geografic Institute) maps for which the respective IGM tablet number have been given.

WS^b^ = Phyto-toponyms available on the web site of the Autonomous Region of Sardinia for which the respective coordinates (WGS84/UTM zone 32 N) have been given.

CM^c^ = Phyto-toponyms in cadastral maps.

SA^d^ = Phyto-toponyms in the State Archive in Cagliari.
